# Validation of a mobile application for adults with neurological lower urinary tract dysfunction*

**DOI:** 10.1590/1518-8345.7145.4323

**Published:** 2024-08-30

**Authors:** Danielle Soraya Lourenço Fernandes Gomes, Priscilla Alfradique de Souza, Gisela Maria Assis, Danielle Galdino de Paula

**Affiliations:** 1 Universidade Federal do Estado do Rio de Janeiro, Rio de Janeiro, RJ, Brazil.; 2 Instituto Nacional de Traumatologia e Ortopedia, Unidade de Estomaterapia, Rio de Janeiro, RJ, Brazil.; 3 Universidade Federal do Estado do Rio de Janeiro, Escola de Enfermagem Alfredo Pinto, Rio de Janeiro, RJ, Brazil.; 4 Universidade Federal do Paraná, Hospital de Clínicas, Curitiba, PR, Brazil.

**Keywords:** Urinary Bladder Neurogenic, Spinal Cord Injury, Intermittent Urethral Catheterization, Health Education, Mobile Applications, Enterostomal Therapy

## Abstract

**Objective:**

to validate with experts the content of a mobile application to guide patients with neurological dysfunction of the lower urinary tract**.**

**Method:**

methodological study. For content validation, 81 specialist nurses took part. The data collection instrument was designed in the form of an electronic form, and included assessments of the application’s objectives, content, language, relevance, functionality and usability. The data was submitted to descriptive and inferential statistical analysis, based on the measurement of the Content Validity Index.

**Results:**

the overall content validity index of the prototype application was 0.98. The lowest item (0.93) was related to the adequacy of the font size, and the highest module (1) was associated with usability, showing ease in handling the application. The experts recommended correcting spelling and grammar, as well as adding and minimizing information, standardizing language, among others.

**Conclusion:**

the content of the *MeduLar sem Medo* ^*®*^ mobile app was validated with excellence by specialists, and presented important resources for teaching urinary dysfunction control and adherence to rehabilitation. With these results, it is possible to envision the next stages of validation, with application in the populations for which it is intended and incorporation of this care technology into the Unified Health System.

## Highlights

**(1)** Innovative study in the nursing teaching-learning process.

**(2)** Innovation within the Unified Health System.

**(3)** Educational tool that is easily accessible to the user.

**(4)** It allows nurses to monitor the learning process.

**(5)** Development of professional skills for health academics.

## Introduction

Spinal cord injury (SCI) is an impacting event, as it affects both the person involved and their family/caregiver. Once the SCI is established, the individual suffers physical and emotional changes which, to varying degrees, can be irreversible or fatal. An example of an irreversible condition is neurological dysfunction of the lower urinary tract^1^ ^)-(^ ^2^.

In research carried out in 2019 by the Sarah Network of Rehabilitation Hospitals by interviewing hospitalized patients, traffic accidents were the leading external cause of hospitalization, with 47.7% of cases. Assaults (including firearms, bladed weapons and physical assault) were the second external cause of hospitalization, accounting for 22.6% of cases. Falls (15.5%), diving accidents (4.5%), impacts by heavy objects (2.7%) and other external causes (7.0%) also appear in this research^3^.

According to the European Association of Urology, the prevalence of traumatic SCI in developed countries ranges from 280 to 906/million^4^.

In a quantitative, retrospective and descriptive study aimed at surveying the epidemiological profile of spinal cord trauma in the Federal District, Brazil, between 2018 and 2020, regarding complications related to spinal cord trauma, the most prevalent were: pressure injuries, pneumonia, atelectasis, urinary tract infection and spasticity^5^.

In people with Lower Urinary Tract Neurological Dysfunction (LUTS), without proper bladder management, vesico-urinary complications are expected, such as repeated urinary infection, bladder stones, peno-urethral fistula, vesico-ureteral reflux, hydronephrosis, even loss of renal function^1^ ^)-(^ ^2^.

Therefore, care for people with urinary dysfunction is essential and should be guided and monitored by a health professional in order to build capacity and strengthen the rehabilitation process. It should be noted that, among the care provided, one should observe the urinary pattern and its characteristics; signal changes in the body and encourage it to rediscover itself in the new condition of life; seek to understand the understanding of urinary dysfunction; provide guidance on performing Clean Intermittent Catheterization (CIC) and behavioral changes; identify risk factors for vesico-urinary complications; present temporary alternatives to incontinence; observe the use of medication; and recognize the acceptance and coping with this new condition^4^.

Researchers carried out a study at a university hospital in Minas Gerais, Brazil, to find out whether nurses working in hospitals caring for people with SCI were prepared to provide CIC guidance. The authors concluded that most of the patients investigated did not receive guidance before being discharged from hospital. It should be noted that the hospital is a reference in SCI care^6^. 

Systematized nursing care should improve patient treatment and prevent complications. In this way, nurses play an important role in health education, in the rehabilitation process and in the social reintegration of people with SCI^6^. 

The use of mobile technologies in health education is proving increasingly relevant and effective these days. Through apps, text messages, videos and other digital tools, it is possible to disseminate information and guidance on health care in an accessible and interactive way. This approach makes it possible to reach a greater number of people, regardless of their geographical location, providing greater engagement in health management. What’s more, mobile technologies make it possible to personalize content according to the needs of each user, making the learning process more effective and adapted to their particularities. Therefore, the integration of health education and mobile technologies represents a powerful tool for nurses in the rehabilitation process. 

Currently, there are no mobile applications (APP) in the national or international literature that provide guidance on the proper management of LUTS, but the following can be found as a source of information for patients with SCI: booklets, infographics, videos and manuals. There are few digital educational tools developed and validated to collaborate in the teaching-learning process^7^. It therefore has innovative potential, as it is a facilitating tool in the teaching-learning process for users, professionals and academics.

Global research into digital health proposes new horizons for health and confirms that mobile technologies are increasingly present in people’s lives and are advancing dynamically and rapidly in society, constituting new educational relationships^8^. The use and expansion of digital health solutions can innovate the way people around the world achieve higher standards of health and access services and/or information to promote and protect health^9^. It should be noted that strengthening digital health is part of the list of priorities of the Digital Health Strategy for Brazil (2020-2028) (ESD28)^9^.

Some of ESD28’s objectives are: support for improving healthcare, user engagement as the protagonist of their own health, a health connectivity environment and an innovation ecosystem^9^.

Considering that mobile technologies can be effective in promoting health in people with LUTS and that there are no apps available in the literature on guidance for people with neurological lower urinary tract dysfunction, this study was proposed.

Thus, the aim of the study was to validate the content of a mobile application by nurse specialists, with a view to guiding adult patients with neurological dysfunction of the lower urinary tract.

## Method

### Type of study

This study is part of the project entitled “*MeduLar sem Medo* ^*®*^: mobile application for adults with neurological dysfunction of the lower urinary tract secondary to traumatic spinal cord injury”. This is a methodological study, in the form of technological innovation to validate an educational application.

The framework used was Design Science Research (DSR), permeated by six stages: the problem/motivation was identified, based on this, the objectives were listed, the product was developed, the testing process was carried out, the experimental evaluation was carried out, through validation by experts in the field, ending with a proposal for scientific dissemination, so that it can be applied in similar situations by various organizations^10^.

DSR is a methodology focused on solving problems identified in a practical context, based on new scientific knowledge applied to the production of products, generally of a technological nature^10^.

The core of this methodology’s guidelines is that the object of the research should be a product; that the problem addressed should be relevant; and that the product should be useful to the user. It requires rigorous evaluation of the product, following systematic research methods; innovative contributions to the research area of knowledge; appropriate use of resources to achieve the desired goals; and communication of the research results to users^10^.

The three initial stages guided by the DSR were developed in a previous study, also permeated by an integrative literature review^11^, with analysis of scientific evidence as the content of the application, guided by the framework of Orem’s Nursing Theory of Self-Care Deficit throughout the construction process. This article presents the content validation stage and the prototype proposition based on the following considerations.

### Study setting

Data was collected in a virtual environment, with national coverage in the five regions of Brazil and internationally (Brazilians living in cities in Colombia, Spain and Portugal).

### Period

Data was collected between March 6 and April 10, 2023.

### Population, inclusion criteria and sample

The population was made up of nurses specializing in stomatherapy or rehabilitation. The inclusion criteria were: having at least a specialist degree in Stomatherapy or Rehabilitation, or at least five years’ experience in the subject. Exclusion criteria: nurses who did not achieve at least five points on the criteria^12^, according to Figure 1.

**Figure 1:** Criteria adapted for selecting the experts in this study. Rio de Janeiro, RJ, Brazil, 2023^12^

CRITERIA	SCORE
Master’s degree in Nursing or a minimum of 5 years’ professional experience	4
Participation in a research group on the subject (>1 year)	1
Publication of an article on the subject in leading journals	2
PhD in Nursing	2
Clinical or teaching experience in the subject (>1 year)	1
Specialization completed in Stomatherapy/Rehabilitation	3

The specialists were first contacted through working groups in the field of urinary dysfunction rehabilitation in Brazil, via e-mail addresses and/or instant messaging applications. The experts were also selected by analyzing their Lattes CVs, available on the Lattes Platform, on the website of the National Council for Scientific and Technological Development (CNPq), using the search terms “urinary incontinence”; “rehabilitation”; “stomatherapy”, “neurogenic bladder”; and “neurological dysfunction of the lower urinary tract”.

Convenience and snowball sampling were also used as recruitment strategies^13^. To compose the panel of experts, more than 300 experts were recruited, of whom 178 agreed to take part and 89 answered the questionnaire, one was excluded for not having completed all the answers to the form and seven experts were excluded for not achieving the minimum 5 points in the criteria^12^. Thus, the final sample consisted of 81 experts.

### Study variables

Socio-demographic variables were chosen (gender, age, length of professional career, highest academic degree, experience in healthcare on the subject, place of work, publication and participation in research groups on the subject). The variables related to the content assessment items were separated into modules: objectives (Module I); content (Module II); language (Module III); relevance (Module IV); functionality (Module V); and usability (Module VI).

### Instrument used for data collection

The data collection instrument was prepared in the form of an online form, using the Google Forms^®^ tool, consisting of seven parts: 1^st^ a brief presentation of the project; 2^nd^ a Free and Informed Consent form; 3^rd^ general guidelines on filling in the form; 4^th^ the experts’ socio-demographic characteristics; 5^th^ a link to the application’s prototype with the items to be evaluated; 6^th^ Access link to the application prototype with the items to be evaluated; 7^th^ Acknowledgement of participation.

### Data collection

The form was sent to the experts by e-mail or messaging application, and information on how to access the prototype was sent by video, showing the screens so that the evaluator could have a similar experience to the user.

### Data analysis

The data was submitted to descriptive and inferential statistical analysis, based on the measurement of the Content Validity Index (CVI). This index consists of measuring the proportion or percentage of experts who agree with the items in the instrument, allowing each item to be analyzed, as well as its entirety^14^ ^)-(^ ^15^.

To analyze the data, a Likert-type scale transformation was considered^12^, in which the following options were rated: completely disagree (value: 0), partially disagree (value: 0.25), neither agree nor disagree (value: 0.50), partially agree (value: 0.75), completely agree (value: 1). For evaluation of both each item and the entire instrument, the CVI values were considered acceptable, starting at 0.50. For each item considered acceptable, it was divided by the number of experts, thus obtaining the proportion of agreement^15^.

To analyze the experts’ overall assessment, the sum of all the CVIs calculated separately was used, divided by the total number of items in the instrument. In the modules, the average CVI was calculated^15^.

Agreement of at least 80% indicates the adequacy of the content, making it pertinent to remain with the product, so for this study, items that obtained CVI ≥ 0.80 were considered valid, so those that obtained values below this threshold would need to be excluded or modified to be re-evaluated^16^. The answers were exported as a document in Microsoft Corporation’s Excel^®^ spreadsheet software and the data was selected, grouped and counted for analysis.

### Ethical aspects

The project was submitted to and approved by the Research Ethics Committee, according to opinion 5.476.943. All the participants in the research were guaranteed confidentiality and anonymity.

## Results

Of the 81 (100%) specialists, 92% were women and their ages ranged from 30 to 73, with the highest frequency between 30 and 40. As for the length of time they had been working, 49% had been working for more than 15 years. With regard to academic qualifications, 55% had completed a specialization course and 84% were stomatherapists. With regard to experience in caring for patients with neurological dysfunction of the lower urinary tract, 63% had more than a year’s experience. However, only 17% had published on the subject of the study and only 23% had participated in groups. 

Regarding characterization according to place of work, all regions of Brazil were covered, with the Central-West Region (5%); North Region (6%); South Region (10%); Northeast Region (17%); and Southeast Region (56%). The states with the highest number of professionals working on this topic were: Rio de Janeiro (n=20/25%), Sao Paulo (n=16/20%), Minas Gerais (n=9/11%) and Ceará (n=7/9%), as well as international cities such as Valencia, Spain; San Cristóbal de La Laguna, Spain; Bogotá, Colombia; and Coimbra, Portugal, as shown in Table 1. International cities refer to Brazilian specialists living abroad.

**Table 1:** Characterization of the application specialists. Rio de Janeiro, RJ, Brazil, 2023

Characteristics	n	%
**Sex**		
Female	75	92
Male	6	8
**Age (years)**		
30 to 40	34	42
41 to 50	31	38
51 to 60	12	15
>60	2	2
**Length of professional career (years)**		
<5	1	1
5-10	21	26
11-15	19	23
>15	40	49
**Higher academic degree**		
Specialization	45	55
Masters	19	23
Doctorate	15	18
Post-doctorate	2	2
**Assistance experience on the subject**		
No	25	31
Yes < 1 year	5	6
Yes > 1 year	51	63
**Location by macro-region**		
North	5	6
North East	14	17
Southeast	45	56
Midwest	4	5
South	8	10
Colombia	1	1
Portugal	1	1
Spain	2	2
**Publication on the subject of the study**		
No	67	83
Yes	14	17
**Participation in a thematic research group**		
No	62	76
Yes > 1 year	19	23
Total	81	100

Regarding the evaluation of the mobile app by the experts, the content validation index by items ranged from 0.93 to 1. The lowest item (0.93) was related to the adequacy of the font size in the app, as the experts considered the font to be small and suggested increasing it.

The maximum score (1) was given to 44% of the items evaluated, with Module II, Content, having the highest concentration of this score, as shown in Table 2.

**Table 2:** Content validation index of the app’s evaluated domains. Rio de Janeiro, RJ, Brazil, 2023

Modules	CVI*
Module I - Objectives	
Item 1- The application prototype meets the proposed objective	0.97
Item 2- The app prototype is suitable for the teaching-learning process	0.98
Item 3- The application prototype clarifies doubts about the topic covered	1
Item 4- The content of the app prototype enables understanding of the topic covered	1
Item 5- The app prototype allows the target audience to reflect on the topic	0.98
Item 6 - The app prototype encourages people to change their habits	0.97
Item 7- The content presented in the app prototype corresponds to the objectives	0.97
proposed in the work	
CVI* Module I	0.98
Module II- Content	
Item 8- The content follows a logical sequence	1
Item 9- The content incorporates all the steps for the CIL^†^ technique in an orderly manner	1
Item 10- The content includes all the materials needed for CIL^†^ guidance	1
Item 11- The information presented in the prototype application is correct	1
Item 12- The information in the prototype application is clear	1
Item 13- The information provided by the app prototype is clear	1
Item 14- The images and videos in the app prototype clearly illustrate the	0.97
presented	0.93
Item 15- The font size of the text is adequate	
CVI* Module II	0.99
Module III- Language	
Item 16- The app prototype presents clear and accessible language	0.96
Item 17- The application prototype presents objective language	0.98
Item 18- The application prototype has interactive language,	0.97
allowing active development in the educational process	
CVI* Module III	0.97
Module IV- Relevance	
Item 19- The app prototype contributes to knowledge of urinary dysfunction	0.98
Item 20- The app prototype arouses interest in the topic	1
Item 21- The app prototype is relevant to the user’s ability to carry out	0.98
safe self-care	
CVI* Module IV	0.99
Module V- Functionality	
Item 22- The application prototype is an appropriate tool	0.98
for its intended purpose	
Item 23- The app prototype is suitable for the intended audience	0.98
Item 24- The application prototype is able to generate positive results	0.98
in the teaching-learning process on the theme	0.98
CVI* Module V	
Module VI- Usability	
Item 25- The application prototype is easy to use	1
Item 26- The application prototype is easy to understand the theoretical concepts used and their applications.	1
Item 27- The application prototype allows the user to easily apply the guidelines provided.	1
CVI* Module VI	1
CVI* Overall	0.98

*CVI= Content Validation Index; ^†^CIL= Clean Intermittent Catheterization

The overall CVI of the APP prototype was 0.98. The items used for the CVI were distributed in the six modules as follows:

1. Objectives, validated in all seven items (CVI=0.98), thus demonstrating the achievement of the goal in relation to the teaching-learning process, covering clarification of doubts, reflection on the topic and encouragement to change habits.
2. Content, validated in all eight items (CVI=0.99), meaning scientific basis presented in a clear and enlightening way.
3. Language, validated in three items (CVI=0.97), was the module with the lowest score, with suggestions about the use of technical terms. However, they mentioned clarity, objectivity, accessibility and interactivity.
4. Relevance, validated on all three items (CVI=0.99), was considered relevant to the user.
5. Functionality, validated on all three items (CVI=0.98), representing suitability for the target audience and being considered a tool for patient education.
6. Usability, validated on all three items (CVI = 1), this module scored highest, expressing ease of handling, easy understanding of the theoretical concepts, since they are technical and complex, as well as providing ease in applying the guidance provided.

The experts who validated the content recommended correcting spelling and grammar, as well as adding and minimizing information and standardizing the language. In addition to answering the instrument’s objective questions, participants made suggestions and comments about the application. The suggestions were taken on board due to their congruence with the literature, as shown in Figure 2.

**Figure 2:** Summary of suggestions and comments from experts. Rio de Janeiro, RJ, Brazil, 2023

Suggestions	Answered
Add catheterization technique with conventional catheter.	Yes
Enable voice command application.	Yes
Replace technical terms.	Yes
Reduce text and increase images.	Yes
Gamification for greater interactivity	Yes
Include social experience.	No
Test with the user.	No
Neurogenic bowel management.	No
Something practical for recording the scale and diaries directly in the app.	No

The relevant suggestions led to changes, corrections and improvements to the application. Others, because they didn’t fit the proposed objective, didn’t lead to changes.

It should be emphasized that the validation by experts took place in just one instance, and there was no need to re-evaluate items.

Specific suggestions were made, the vast majority of which were met. Module I, which refers to the objective, received the most suggestions, with the aim of being more dynamic, interactive and with more information, including more images, videos and instructions recorded in the author’s voice.

For illiterate users or those with compromised visual acuity, it was suggested that voice commands should be enabled for the application. Based on the experts’ suggestions, the language was revised and new images and audio were produced to replace the text. 

To improve interactivity with the user, a gamification strategy was developed, using a quiz categorized by subject, providing the user with a knowledge assessment tool. In order to encourage changes in habits and reintegration into society, the *MeduLar sem Medo* ^*®*^ app also included topics on stimuli for social interaction. 

As for the suggestion of testing with the user, although it is important, it has not been defined as part of this stage of the research, it will be part of future studies, and records directly in the app are not covered at this time, as this would require cloud storage and would be more expensive. However, the aim is to make progress in this area within the app.

With regard to the comments reported by the experts on the *MeduLar sem Medo* ^*®*^ app, they confirm that it has been validated with excellence. They stand out:

- To be an excellent tool for quick and easy support for people with SCI, helping to clarify doubts and identifying what could be improved in their care.
- It has the necessary resources for active self-care, helping with treatment adherence, with factors that promote changes in habits and, consequently, the prevention of complications.
- To disseminate in Primary Care and health services, adding the possibility for nurses to improve and monitor the learning process of people with SCI.

## Discussion

The *MeduLar sem Medo* ^*®*^ application was validated by experts with a global CVI of 0.98, which corresponds to excellent content validity^15^, indicating that experts are in statistically significant agreement on the content of the mobile app. 

This fact demonstrates that the application reveals an understanding of the proposed topic, follows a logical sequence and incorporates the organization of the material and the technique of clean intermittent catheterization in an orderly manner, which makes it possible to clarify doubts. Furthermore, it provides information with accurate, clear and enlightening content, in addition to being interactive, which can arouse the user’s interest in the topic. 

Validation by experts with extensive experience, in addition to significant academic training, enabled adaptation and improvement of the application and a careful evaluation and credibility of the technology in the final version. The work of specialists in different regions allows the adaptation of the product built to the general context, considering the cultural diversity of continental dimensions.

Regarding professional occupation, there was a predominance of nurses working directly with assistance on the subject, that is, who directly assist the person with SCI, knowing the weaknesses in care. It is noteworthy that, based on this study, the participation of experts publishing articles on the subject and belonging to research groups was low. 

Module I - Objectives, showed potential in the teaching-learning process, in addition to providing reflection for the person with SCI and encouraging changes in habits. Scholars evaluated a mobile application, based on the experience of pregnant women, accompanied in prenatal consultations in the public health sector. It was observed that the application was a favorable tool to help improve and build new knowledge and improve self-care, when encouraged by a professional^17^.

In another study, the authors developed and validated a mobile application aimed at pregnant adolescent women in primary health care. The application was positively evaluated both in quality and usability, in which it was concluded that the creation of the digital resource presents itself as a complementary and supportive way to the process of educating pregnant women^18^.

Another mobile application on nursing care for people with diabetes mellitus demonstrated the potential to assist professional nurses in tracking, monitoring and preventing complications in users with diabetes^19^.

When analyzing the effectiveness of the intervention led by nurses, the benefits of the mobile application for mothers with depression were verified. The intervention was effective and the application was easy to use and considered a complement to existing support services for mothers with depression^20^.

Evidence from research with similar proposals corroborates how an application has the potential to be an educational tool and encourages self-care. From this perspective, Module II, content, proved to be enlightening and correct in terms of information. Thus, the product presents scientific information based on an integrative review and with a theoretical framework with singular attention to the person with SCI. It is noteworthy that adherence to self-care is complex, rehabilitation requires mastery of specific technical knowledge and the nurse’s understanding of the phases of acceptance by the patient of their own condition.

The professional’s mastery of understanding in the phases of denial, repercussion, adjustment and reconstruction to the new performance, with a focus on overcoming, interaction and social inclusion, is essential for successful rehabilitation^21^. The application has motivational strategies for those in the denial phase and educational strategies for those in the adjustment and/or reconstruction phase, with a potential impact on improving the person with LUTS. In this way, we hope to contribute to better coping at each stage.

In a qualitative study carried out in 2020, in the city of Natal, Rio Grande do Norte, Brazil, with the objective of understanding the knowledge and practices of primary care professionals regarding the care of people with SCI, the authors reported that the care of people with SCI is permeated by structural difficulties, above all, with a lack of professional training for comprehensive care, with emphasis on the need for ongoing education actions to improve care^22^.

Thus, it is observed that the *MeduLar sem Medo* ^*®*^ application has the potential to transform the teaching-learning process, being a complementary tool, as it expands the integration of the professional with the person with SCI, with a view to achieving comprehensive care, due to the lack of professional training. It also constitutes an instrument of ongoing education in health institutions. 

The nurse who monitors the progress of confrontations and awakens the motivation of people with disabilities to take care of themselves and recognize the need for help with self-care, must be ready to enter this stage of treatment, as they find a suitable space to invest in education in health and collaborate in the rehabilitation and autonomy of the patient^21^.

The content regarding guidance regarding the conventional catheter, suggested by the specialist, was met. Although the gold standard is the hydrophilic catheter, this is not the reality for many users. It is the right guaranteed by law that the individual who performs intermittent catheterization receives the material to perform it. Ordinance No. 37, of July 24, 2019, article 1, establishes the hydrophilic catheter for intermittent catheterization in individuals with SCI, as established by the Ministry of Health, within the scope of the Unified Health System (SUS). Thus, ensuring access to the material necessary for the procedure is part of the health treatment of these individuals, and it is therefore the SUS’s duty to ensure availability. However, each municipality has autonomy to establish the release flow of this material^23^. When there are difficulties that make it impossible for the patient to access this material, then a conventional catheter is available. 

Concerning the reference to Module III - Language - this proved to contain clear, accessible and interactive language. In an integrative review carried out, considering the period from 2010 to 2020, to evaluate mobile applications developed and implemented in children and adolescents with chronic diseases, it was identified that the use of humor, gamification and simple language and attractive visuals aroused interest and facilitated use of the application and also favored adherence to self-care^24^.

Interaction with the user is relevant, as it is through this that the application will fulfill its educational and motivational objective. The interaction strategies went beyond simple language but also in the format of dialogue. Technological resources such as images, audios and videos were used in order to make the application more attractive and less tiring. Another strategy used was gamification, which can help the user to measure the knowledge acquired. Furthermore, the use of informative audios recorded by the researcher is expected to raise awareness about self-care.

Module IV - Relevance - proved to be relevant in the self-care process with reflection and action strategies. Furthermore, the main laws that deal with the rights of people with SCI were sought, with the aim of providing information, through a link that will direct to the official website.

In relation to Module V - Functionality - it demonstrated a learning function for the person with LUTS and also assistance to the professional who will carry out the hospital discharge process or the professional who will monitor rehabilitation^21^.

Module VI - Usability - proved to be simple and intuitive to use, in addition to providing knowledge of complex concepts for understanding, facilitating the understanding of the new urinary physiology of people with SCI and the respective complications^21^. 

The purpose of this technology is to include the person with SCI in the treatment itself. Health is defined as “a complete state of physical, mental and social well-being, and does not consist merely of the absence of illness or disease”^25^. According to the health system, the professional must be prepared to listen to the user, understand the social context in which they are inserted and, from then on, meet the demands and needs, paying attention, above all, to the prevention of health problems^25^. One of the essential measures to improve the survival, health and participation of people with SCI is access to continuous health care and health education^26^.

Ordinance No. 793 of 2012, established the Care Network for Persons with Disabilities, within the scope of the Unified Health System, chapter I, article 2, in the general provisions, is among other guidelines for the operation, the promotion of health strategies continuing education, technological innovation in rehabilitation, linked to the actions of the National Center for Assistive Technology, use of free technologies for management, mainly focused on information areas^27^. 

In articles 3 and 4, with the objectives of the Care Network, it is highlighted to expand access and qualify care for people with disabilities in the SUS, promote permanent training mechanisms for health professionals and produce and offer information on people’s rights, prevention and care measures and services available from the network, through notebooks, booklets and manuals^27^.

In chapter II, article 11, the components of the Care Network are presented, which are organized into: I- Primary Care; II- Specialized Rehabilitation Care (CER); III- Hospital Care, whose components of the Network will be articulated with each other, in order to guarantee comprehensive care and regulated access to each point of care and/or services, which are, among others: clinical management and measures to prevent functional loss, reduction in the rate of functional loss and/or improvement or recovery of function^27^.

There is evidence about the global need and access to assistive products, and it provides a series of recommendations to expand availability and access. The report recommends, among others, improving access to education, health and social assistance systems, guaranteeing the availability, safety, effectiveness and accessibility of assistive products and active involvement of users of assistive technology and their families^28^.

Assistive technology is an umbrella term for assistive products and related systems and services. Assistive products are capable of improving performance in the main functional domains, such as self-care, and can be physical products, such as a wheelchair or software and digital applications^28^.

That said, the *MeduLar sem Medo* ^*®*^ application presents specialized qualifications and education and motivation topics to contribute to the Care Networks for Persons with Disabilities, within the scope of the SUS, in order to promote health, in addition to contributing to the training of healthcare professionals. health on this topic.

This study is expected to contribute to learning innovation in care, education and teaching. Finally, the mobile application was registered with the National Institute of Industrial Property (INPI), under the domain of the Federal University of Rio de Janeiro, with free access to the user.

As limitations of the study, it refers to the non-validation of this technology by the person with SCI, in terms of usability and applicability, that is, the impact on improving adherence to self-care, which is configured as a proposal for continuity. And, also, the fact that the application does not have user information stored in the cloud, which is being evaluated for future possibilities.

## Conclusion

The *MeduLar sem Medo* ^*®*^ app was validated by experts in terms of its content for guiding people with neurological dysfunction of the lower urinary tract, with an excellent overall rating, which demonstrates its accuracy for the population.

Future implications include the promotion of health education for people with LUTS, in order to encourage self-care with technical information and also by promoting adherence to care, through some strategies, motivating changes in habits.

It is also intended to collaborate with the training of professionals and contribute to the learning of health academics, bringing them closer to this subject at an early stage. 

As a future research proposal, the mobile application could be applied to the populations for which it is intended, with a view to incorporating this care technology into the Unified Health System through the Care Network, whether primary or specialized care, in order to help reduce secondary complications and reduce costs for the health system, through education.
