# Supplementary material for: Validation of a mobile application for adults with neurological lower urinary tract dysfunction
Source: Rev Lat Am Enfermagem. 2024 Aug 30;32:e4323. doi: 10.1590/1518-8345.7145.4323 (PMC11368066; doi:10.1590/1518-8345.7145.4323)
Supplement: Translation

# Translation: Validação de aplicativo móvel para adultos com disfunção neurológica do trato urinário inferior*

**DOI:** 10.1590/1518-8345.7145.4324

**Authors:** Danielle Soraya Lourenço Fernandes Gomes, Priscilla Alfradique de Souza, Gisela Maria Assis, Danielle Galdino de Paula

**Affiliations:** 1 Universidade Federal do Estado do Rio de Janeiro, Rio de Janeiro, RJ, Brasil.; 2 Instituto Nacional de Traumatologia e Ortopedia, Unidade de Estomaterapia, Rio de Janeiro, RJ, Brasil.; 3 Universidade Federal do Estado do Rio de Janeiro, Escola de Enfermagem Alfredo Pinto, Rio de Janeiro, RJ, Brasil.; 4 Universidade Federal do Paraná, Hospital de Clínicas, Curitiba, PR, Brasil.

**Keywords:** Bexiga Urinaria Neurogênica, Traumatismos da Medula Espinal, Cateterismo Uretral Intermitente, Educação em Saúde, Aplicativos Móveis, Estomaterapia

## Abstract

**Objetivo:**

validar com especialistas o conteúdo de aplicativo móvel para orientação de pacientes com disfunção neurológica do trato urinário inferior**.**

**Método:**

estudo metodológico. Para validação do conteúdo, participaram 81 enfermeiros especialistas. O instrumento de coleta de dados foi elaborado no formato de formulário eletrônico e conteve avaliações sobre objetivos, conteúdo, linguagem, relevância, funcionalidade e usabilidade do aplicativo. Os dados foram submetidos à análise estatística descritiva e inferencial, com base na mensuração do Índice de Validade de Conteúdo.

**Resultados:**

o índice de validade de conteúdo global do protótipo de aplicativo foi de 0,98. O item mais baixo (0,93) foi referente à adequação do tamanho da fonte, e o módulo mais alto (1) foi associado à usabilidade, que mostrou facilidade no manuseio do aplicativo. Os especialistas recomendaram correção ortográfica e gramatical, além de acréscimos e minimização de informações, padronização da linguagem, dentre outros.

**Conclusão:**

o conteúdo do aplicativo móvel MeduLar sem Medo^®^ foi validado com excelência por especialistas, pois apresentou recursos importantes para o ensino do controle da disfunção urinária e a adesão à reabilitação. Com tais resultados, é possível vislumbrar as próximas etapas de validação, com aplicação nas populações a que se destina e incorporação desta tecnologia de cuidado no âmbito do Sistema Único de Saúde.

## Destaques

**(1)** Estudo inovador no processo de ensino-aprendizagem de enfermagem.

**(2)** Inovação no âmbito do Sistema Único de Saúde.

**(3)** Ferramenta de educação de fácil acesso ao usuário.

**(4)** Proporciona ao enfermeiro o acompanhamento do processo de aprendizagem.

**(5)** Desenvolvimento de competências profissionais aos acadêmicos da saúde.

## Introdução

A Lesão Medular (LM) é um evento impactante, uma vez que afeta a pessoa envolvida e a família/cuidador. Após a instalação da LM, o indivíduo sofre alterações físicas e emocionais que, em graus variados, podem ser irreversíveis ou fatais. Exemplo de condição irreversível é a disfunção neurológica do trato urinário inferior^1^ ^)-(^ ^2^.

Pesquisa realizada em 2019, pela Rede Sarah de Hospitais de Reabilitação, por meio de entrevista em pacientes internados, mostrou que os acidentes de trânsito foram a primeira causa externa de internação, com 47,7% dos casos. As agressões (incluindo arma de fogo, arma branca e agressão física) constituem a segunda causa externa de internação, 22,6% dos casos. Ainda aparecem como causas externas, nessa mesma pesquisa, as quedas, 15,5%; os acidentes com mergulho, 4,5%; os impactos por objetos pesados, 2,7%; entre outras, 7,0%^3^. E, de acordo com *European Association of Urology*, a prevalência de LM traumática em países desenvolvidos varia de 280 a 906/milhões^4^.

Todavia, em estudo quantitativo, retrospectivo e descritivo com objetivo de levantamento do perfil epidemiológico do trauma raquimedular no âmbito do estado do Distrito Federal, Brasil, entre os anos de 2018 e 2020, ao se referir às complicações atinentes do trauma raquimedular, os mais prevalentes foram: lesões por pressão, pneumonia, atelectasia, infecção do trato urinário e espasticidade^5^.

Ainda, entende-se que em pessoas com Disfunção Neurológica do Trato Urinário Inferior (DNTUI), sem o manejo adequado da bexiga, são esperadas complicações vesico-urinárias, como infecção urinária de repetição, cálculo vesical, fístula peno-uretral, refluxo vesico ureteral, hidronefrose e até perda da função renal^1^ ^)-(^ ^2^.

Logo, cuidados às pessoas com disfunção urinária são essenciais e devem ser orientados e acompanhados por profissional de saúde, a fim de criar capacidade e fortalecer o processo de reabilitação. Destaca-se que, dentre os cuidados, deve-se observar o padrão urinário e as respectivas características; sinalizar mudanças no organismo e incentivá-lo a se redescobrir na nova condição de vida; buscar compreender o entendimento sobre a disfunção urinária; orientar quanto à realização do Cateterismo Intermitente Limpo (CIL) e às mudanças comportamentais; identificar fatores de risco para as complicações vesico-urinárias; apresentar alternativas temporárias à incontinência; observar o uso de medicamentos; e reconhecer a aceitação e o enfrentamento desta nova condição^4^.

Posto isto, pesquisadores realizaram estudo em Hospital Universitário, situado em Minas Gerais, Brasil; e investigaram se os enfermeiros atuantes em hospitais em atendimento à pessoa com LM estão preparados para orientar o CIL. Os autores concluíram que a maioria dos pacientes investigados não recebeu orientação antes da alta hospitalar. Cabe destacar que o hospital é referência no atendimento à LM^6^. Vale salientar que a assistência de enfermagem sistematizada deve promover a melhoria do tratamento do paciente e a prevenção de complicações. Deste modo, o enfermeiro desempenha papel importante na educação em saúde, no processo de reabilitação e na reinserção social de pessoas com LM^6^. 

Em contrapartida, a utilização de tecnologias móveis na educação em saúde tem se mostrado cada vez mais relevante e eficaz nos dias de hoje. Através de aplicativos, mensagens de texto, vídeos e outras ferramentas digitais é possível divulgar informações e orientações sobre cuidados com a saúde de forma acessível e interativa. Essa abordagem permite alcançar um maior número de pessoas, independentemente de sua localização geográfica, proporcionando um maior engajamento na gestão da saúde. Além disso, as tecnologias móveis possibilitam a personalização do conteúdo de acordo com as necessidades de cada usuário, tornando o processo de aprendizado mais eficaz e adaptado às particularidades. Portanto, a integração entre educação em saúde e tecnologias móveis representa uma ferramenta poderosa ao enfermeiro no processo de reabilitação. 

Atualmente não há aplicativos móveis (APP) referido na literatura nacional e internacional que orientem sobre o adequado manejo da DNTUI, apenas encontram-se como fonte de informação para pacientes com LM: cartilhas, infográficos, vídeos e manuais. Ainda são poucas as ferramentas educacionais digitais desenvolvidas e validadas para colaborar no processo ensino-aprendizagem^7^. Apresenta-se, portanto, um potencial inovador, por se tratar de ferramenta facilitadora no processo de ensino-aprendizagem aos usuários, profissionais e acadêmicos.

À vista disto, uma pesquisa global sobre saúde digital propõe novos horizontes para a saúde e ratifica que as tecnologias móveis estão cada vez mais presentes na vida das pessoas e avançam de forma dinâmica e veloz na sociedade, constituindo novas relações educacionais^8^. Ou seja, o uso e a ampliação de soluções digitais de saúde podem inovar a forma como as pessoas no mundo alcançam padrões mais elevados de saúde e acessam serviços e/ou informações para promover e proteger a saúde^9^. Além disso, destaca-se que o fortalecimento em saúde digital faz parte do rol de prioridades da Estratégia de Saúde Digital para o Brasil (2020-2028) (ESD28)^9^. Dentre os objetivos da ESD28 destacam-se o suporte à melhoria da atenção à saúde, o engajamento do usuário como protagonista da própria saúde, o ambiente de conectividade em saúde e o ecossistema de inovação^9^.

Ao considerar que as tecnologias móveis podem ser eficientes para promoção da saúde em pessoas com DNTUI e que não há na literatura aplicativos disponíveis sobre orientação às pessoas com disfunção neurológica do trato urinário inferior, propôs-se o presente estudo. Assim, objetivou-se validar o conteúdo de um aplicativo móvel por especialistas enfermeiros, com vistas à orientação de pacientes adultos com disfunção neurológica do trato urinário inferior.

## Método

### Tipo de estudo

Este estudo compõe o projeto intitulado “MeduLar sem Medo^®^: aplicativo móvel para adultos com disfunção neurológica do trato urinário inferior secundária à lesão medular traumática”. E trata-se de estudo metodológico, na modalidade inovação tecnológica para validação de aplicativo educacional.

Além do mais, o referencial utilizado foi o *Design Science Research* (DSR), permeado pelas seis etapas: identificou-se o problema/motivação; a partir disto, elencaram-se os objetivos; desenvolveu-se o produto; executou-se o processo de testagem; efetivou-se a avaliação experimental, por meio da validação por especialistas na área; finalizando com proposta de divulgação científica, a fim de que seja aplicado em situações similares por diversas organizações^10^.

O DSR se trata de metodologia focada na resolução de problemas identificados no contexto prático, a partir de novos conhecimentos científicos aplicados à produção de produtos, geralmente de natureza tecnológica^10^. Essa metodologia tem como cerne das diretrizes o objeto de pesquisa como produto; relevância do problema abordado e utilidade do produto para o usuário. E, ainda, exige avaliação rigorosa do produto, seguindo os métodos sistemáticos de pesquisa; contribuições inovadoras para área de conhecimento da pesquisa; uso adequado dos recursos para se alcançar as metas desejadas; e comunicação dos resultados da pesquisa aos usuários^10^.

Nesse ínterim, as três etapas iniciais orientadas pelo DSR foram desenvolvidas em estudo anterior, permeada também por uma revisão integrativa da literatura^11^, com análise de evidências científicas como conteúdo do aplicativo, guiado pelo referencial da Teoria de Enfermagem do Déficit do Autocuidado de Orem, em todo processo de construção. Neste artigo apresentam-se a etapa de validação de conteúdo e a proposição do protótipo a partir dessas considerações.

### Local do estudo

A coleta de dados foi realizada em ambiente virtual, com abrangência nacional nas cinco regiões do Brasil e internacional (brasileiros residentes em cidades da Colômbia, Espanha e Portugal).

### Período

A coleta de dados foi realizada entre 6 de março e 10 de abril de 2023.

### População, critérios de inclusão e amostra

A população foi constituída por enfermeiros especialistas em estomaterapia ou reabilitação. Os critérios de inclusão foram: ter, no mínimo, título de especialista em Estomaterapia ou Reabilitação, ou experiência mínima de cinco anos na mesma área. Os critérios de exclusão: enfermeiros que não atingissem, no mínimo, cinco pontos nos critérios^12^, de acordo com a Figura 1.

**Figura 1:** Critérios adaptados para seleção dos peritos deste estudo. Rio de Janeiro, RJ, Brasil, 2023^12^

CRITÉRIOS	PONTUAÇÃO
Titulação de Mestre em Enfermagem ou mínimo de 5 anos de atuação profissional	4
Participação em Grupo de Pesquisa na temática (>1 ano)	1
Publicação de artigo sobre a temática em periódicos de referência	2
Titulação de Doutor em Enfermagem	2
Experiência clínica ou de ensino na temática (>1 ano)	1
Especialização concluída em Estomaterapia/Reabilitação	3

O contato com os especialistas foi primeiramente em grupos de trabalho da área de reabilitação de disfunção urinária no Brasil, por meio de endereços eletrônicos (*e-mail*) e/ou aplicativo de mensagem instantânea. Também se realizou, para seleção dos especialistas, a análise do Currículo Lattes, disponibilizado na Plataforma Lattes, pelo Portal do Conselho Nacional de Desenvolvimento Científico e Tecnológico (CNPq), utilizando-se para busca os termos “incontinência urinária”; “reabilitação”; “estomaterapia”, “bexiga neurogênica”; e “disfunção neurológica do trato urinário inferior”.

Além disso, também como estratégia de recrutamento, aplicou-se a amostragem por conveniência e bola de neve^13^. Para composição do painel de especialistas, foram recrutados mais de 300 especialistas, dos quais 178 aceitaram participar e 89 responderam ao questionário. Um foi excluído por não ter completado todas as respostas do formulário e, ainda, por critério de exclusão, sete especialistas foram excluídos por não atingirem o mínimo de 5 pontos nos critérios^12^. Assim, a amostra final foi constituída por 81 especialistas.

### Variáveis do estudo

Elegeram-se as variáveis sociodemográficas (sexo, idade, tempo de atuação profissional, maior titulação acadêmica, experiência assistencial na temática, local de atuação, publicação e participação em grupos de pesquisa na temática). As variáveis relacionadas aos itens de avaliação de conteúdo foram separadas por módulos: objetivos (Módulo I), conteúdo (Módulo II), linguagem (Módulo III), relevância (Módulo IV), funcionalidade (Módulo V) e usabilidade (Módulo VI).

### Instrumento utilizado para coleta de dados

O instrumento de coleta de dados foi elaborado no formato de formulário *online*, utilizando-se da ferramenta *Google Forms* ^*®*^ e composto de sete partes: 1ª. composta por breve apresentação do projeto; 2ª. Termo de Consentimento Livre e Esclarecido; 3ª. orientações gerais quanto ao preenchimento do formulário; 4ª. caracterização sociodemográfica dos especialistas; 5ª. caracterização profissional dos especialistas; 6ª. *link* de acesso ao protótipo do aplicativo com os itens a serem avaliados; 7ª. agradecimento pela participação.

### Coleta de dados

Enviou-se o formulário aos especialistas por e-mail ou aplicativo de mensagens. Em contrapartida, as informações para o acesso ao protótipo foram exibidas por meio de vídeo, apresentando as telas de forma que o avaliador pudesse ter experiência similar à do usuário.

### Análise de dados

Os dados foram submetidos à análise estatística descritiva e inferencial, com base na mensuração do Índice de Validade de Conteúdo (IVC). Este índice consiste na mensuração da proporção ou porcentagem de especialistas que estão em concordância sobre os itens do instrumento, permitindo analisar cada item, bem como a integralidade dele^14^ ^)-(^ ^15^.

Por conseguinte, para análise dos dados, considerou-se a transformação da escala do tipo Likert^12^, em que foram atribuídas as classificações para opções: discordo completamente (valor: 0), discordo parcialmente (valor: 0,25), não concordo e nem discordo (valor: 0,50), concordo parcialmente (valor: 0,75) e, por fim, concordo totalmente (valor: 1). Para avaliação, tanto de cada item quanto para todo o instrumento, os valores para IVC foram considerados aceitáveis a partir de 0,50. Para cada item considerado aceitável, foi dividido pelo número de especialistas, assim, obtendo-se a proporção de concordância^15^. E, para análise da avaliação global dos especialistas, utilizou-se do somatório de todos os IVC calculados separadamente, dividido pelo número total de itens do instrumento. Nos módulos, calculou-se a média do IVC^15^.

Nessa conjuntura, a concordância de pelo menos 80% indica a adequação do conteúdo, sendo pertinente a permanência no produto. Assim, para este estudo, considerou-se como válido os itens que obtiveram IVC ≥ 0,80. Logo, os que obtiveram valores inferiores a este limiar precisariam ser excluídos ou modificados para serem reavaliados^16^. As respostas foram exportadas em forma de documento no *software* de planilhas *Excel* ^*®*^ , da *Microsoft Corporation*, e os dados foram selecionados, agrupados e contabilizados para análise.

### Aspectos éticos

O projeto foi submetido e aprovado junto ao Comitê de Ética em Pesquisa, conforme parecer 5.476.943. A todos os participantes da pesquisa, garantiram-se o caráter sigiloso dos dados e o anonimato.

## Resultados

Do total de 81 (100%) especialistas, 92% eram mulheres, e a idade variou de 30 a 73 anos, sendo a maior frequência entre 30 e 40 anos. Quanto ao tempo de atuação profissional, 49% apresentaram tempo superior a 15 anos. Em relação à titulação acadêmica, 55% tinham cursado a especialização, sendo que 84% eram estomaterapeutas. Quanto à experiência assistencial a pacientes com disfunção neurológica do trato urinário inferior, 63% tinham experiência superior há um ano. Entretanto, apenas 17% tinham publicação na temática do estudo e somente 23% participaram em grupos de pesquisa na temática. 

No tocante à caracterização segundo o local de atuação, foram contempladas todas as regiões do Brasil, sendo da Região Centro-Oeste (5%), Região Norte (6%), Região Sul (10%), Região Nordeste (17%) e Região Sudeste (56%). Os estados com maior número de profissionais de atuação nesta temática foram: Rio de Janeiro (n=20/25%), São Paulo (n=16/20%), Minas Gerais (n=9/11%) e Ceará (n=7/9%); e, ainda, cidades internacionais, como Valencia, Espanha; San Cristóbal de La Laguna, Espanha; Bogotá, Colômbia; e Coimbra, Portugal, conforme Tabela 1. As cidades internacionais se referem a especialistas brasileiros que residem no exterior.

**Tabela 1:** Caracterização dos especialistas do aplicativo. Rio de Janeiro, RJ, Brasil, 2023

Características	n	%
**Sexo**		
Feminino	75	92
Masculino	6	8
**Idade (anos)**		
30 a 40	34	42
41 a 50	31	38
51 a 60	12	15
>60	2	2
**Tempo de atuação profissional (anos)**		
<5	1	1
5-10	21	26
11-15	19	23
>15	40	49
**Maior titulação acadêmica**		
Especialização	45	55
Mestrado	19	23
Doutorado	15	18
Pós-doutorado	2	2
**Experiência assistencial na temática**		
Não	25	31
Sim < 1 ano	5	6
Sim > 1 ano	51	63
**Local de atuação por macrorregião**		
Norte	5	6
Nordeste	14	17
Sudeste	45	56
Centro-Oeste	4	5
Sul	8	10
Colômbia	1	1
Portugal	1	1
Espanha	2	2
**Publicação na temática do estudo**		
Não	67	83
Sim	14	17
**Participação em grupo de pesquisa temática**		
Não	62	76
Sim > 1 ano	19	23
Total	81	100

Quanto à avaliação do aplicativo móvel pelos especialistas, o índice de validação de conteúdo por itens variou entre 0,93 e 1. O item mais baixo (0,93) foi referente à adequação do tamanho da fonte no aplicativo, pois os especialistas consideraram a fonte pequena, sugerindo o aumento dela. Já a pontuação máxima (1) foi apresentada em 44% dos itens avaliados, tendo o Módulo II, Conteúdo, a maior concentração desta pontuação (Tabela 2).

**Tabela 2:** Índice da validação de conteúdo dos domínios avaliados do aplicativo. Rio de Janeiro, RJ, Brasil, 2023

Módulos	IVC*
Módulo I - Objetivos	
Item 1- O protótipo do aplicativo contempla o objetivo proposto	0,97
Item 2- O protótipo do aplicativo é adequado ao processo ensino-aprendizagem	0,98
Item 3- O protótipo do aplicativo esclarece dúvidas sobre o tema abordado	1
Item 4- O conteúdo do protótipo do aplicativo permite a compreensão do tema abordado	1
Item 5- O protótipo do aplicativo proporciona reflexão do público-alvo sobre o tema	0,98
Item 6- O protótipo do aplicativo incentiva a mudança de hábitos	0,97
Item 7- O conteúdo apresentado no protótipo do aplicativo corresponde aos objetivos propostos no trabalho	0,97
IVC* Módulo I	0,98
Módulo II- Conteúdo	
Item 8- O conteúdo obedece a uma sequência lógica	1
Item 9- O conteúdo incorpora, de forma ordenada, todos os passos para a técnica do CIL^†^	1
Item 10- O conteúdo dispõe de todos os materiais necessários para a orientação do CIL^†^	1
Item 11- As informações que o protótipo do aplicativo apresenta estão corretas	1
Item 12- As informações que o protótipo do aplicativo apresenta estão claras	1
Item 13- As informações que o protótipo do aplicativo apresenta são esclarecedoras	1
Item 14- As imagens e os vídeos do protótipo do aplicativo ilustram de forma clara os conteúdos apresentados	0,97
Item 15- O tamanho da fonte do texto está adequado	0,93
IVC* Módulo II	0,99
Módulo III- Linguagem	
Item 16- O protótipo do aplicativo apresenta linguagem clara e acessível	0,96
Item 17- O protótipo do aplicativo apresenta linguagem objetiva	0,98
Item 18- O protótipo do aplicativo possui linguagem interativa, permitindo o desenvolvimento ativo no processo educativo	0,97
IVC* Módulo III	0,97
Módulo IV- Relevância	
Item 19- O protótipo do aplicativo contribui para o conhecimento da disfunção urinária	0,98
Item 20- O protótipo do aplicativo faz despertar interesse pelo tema	1
Item 21- O protótipo do aplicativo é relevante para que o usuário possa realizar o autocuidado seguro	0,98
IVC* Módulo IV	0,99
Módulo V- Funcionalidade	
Item 22- O protótipo do aplicativo apresenta-se como ferramenta adequada para o objetivo a que se destina	0,98
Item 23- O protótipo do aplicativo apresenta-se adequado para o público destinado	0,98
Item 24- O protótipo do aplicativo possibilita gerar resultados positivos no processo ensino-aprendizagem na temática	0,98
IVC* Módulo V	0,98
Módulo VI- Usabilidade	
Item 25- O protótipo do aplicativo é fácil de usar	1
Item 26- O protótipo do aplicativo é de fácil compreensão dos conceitos teóricos utilizados e suas aplicações.	1
Item 27- O protótipo do aplicativo permite que o usuário tenha facilidade em aplicar as orientações fornecidas.	1
IVC* Módulo VI	1
IVC* Global	0,98

*IVC= Índice de Validação de Conteúdo; ^†^CIL= Cateterismo Intermitente Limpo

O IVC global do protótipo de APP foi 0,98. Os itens utilizados para o IVC foram distribuídos nos seis módulos da seguinte forma:

1. Objetivos, validado nos sete itens (IVC=0,98), assim, mostrando o alcance da meta em relação ao processo ensino-aprendizagem, abrangendo esclarecimentos de dúvidas, reflexão sobre o tema e incentivo às mudanças de hábitos;
2. Conteúdo, validado nos oito itens (IVC=0,99), significando embasamento científico apresentado de forma clara e esclarecedora;
3. Linguagem, validado nos três itens (IVC=0,97), foi o módulo com menor pontuação, pois apresentou sugestões sobre o uso de termos técnicos. Todavia, mencionaram clareza, objetividade, acessibilidade e interatividade;
4. Relevância, validado nos três itens (IVC=0,99), foi considerado relevante ao usuário;
5. Funcionalidade, validado nos três itens (IVC=0,98), representando adequação ao público-alvo e sendo considerada ferramenta para a educação do paciente;
6. Usabilidade, validado nos três itens (IVC= 1), este módulo apresentou maior pontuação, exprimindo a compreensibilidade no manuseio, a fácil assimilação dos conceitos teóricos, visto que são técnicos e complexos, além de proporcionar a facilidade na aplicação das orientações fornecidas.

Vale frisar que os especialistas que validaram o conteúdo recomendaram correção ortográfica e gramatical, além de acréscimos e minimização de informações e padronização da linguagem. Ademais, os participantes que responderam às perguntas objetivas do instrumento, fizeram sugestões e comentários a respeito do aplicativo. As sugestões foram atendidas em decorrência de congruência com a literatura (Figura 2).

**Figura 2:** Síntese das sugestões e dos comentários dos especialistas. Rio de Janeiro, RJ, Brasil, 2023

Sugestões	Atendida
Acrescentar técnica de cateterismo com cateter convencional.	Sim
Habilitar aplicativo para comando de voz.	Sim
Substituir termos técnicos.	Sim
Redução de texto e aumento de imagem.	Sim
Gamificação para maior interatividade	Sim
Incluir vivência social.	Sim
Testar com o usuário.	Não
Manejo do intestino neurogênico.	Não
Algo prático para registro da escala e dos diários direto no aplicativo.	Não

As sugestões julgadas pertinentes conduziram para alterações, correções e aperfeiçoamento do aplicativo. Outras, por não se adequarem ao objetivo proposto, não geraram modificações. Enfatiza-se que a validação por especialistas ocorreu em apenas um momento, não havendo necessidade de reavaliação de itens. 

Procederam-se às sugestões específicas, em que a grande maioria foram atendidas. O Módulo I, referente ao objetivo, foi o que apresentou o maior número de sugestões, com o propósito de ser mais dinâmico, interativo e com mais informações, incluindo mais imagens, vídeos e instruções com gravações na voz da autora.

Além do mais, foi sugerido para usuários analfabetos, ou com acuidade visual comprometida, a habilitação de comando de voz ao aplicativo. A partir da sugestão dos especialistas, realizou-se revisão de linguagem e produziram-se novas imagens e áudios que substituíram textos. 

Ainda, para melhorar a interatividade com o usuário, construiu-se estratégia de gamificação, por meio de *quiz* categorizado por assunto, fornecendo ao usuário ferramenta de avaliação do conhecimento. Com o propósito de incentivar mudanças de hábito e de reintegrar à sociedade, também foi incluído no aplicativo MeduLar sem Medo^®^ tópicos sobre estímulos para convivência social. 

Quanto à sugestão de testar com o usuário, apesar da sua importância, não se definiu como parte desta etapa da pesquisa e integrará estudos futuros. Também não sendo atendidos neste estudo, registros diretamente no aplicativo, pois para esta função, necessitaria de armazenamento em nuvem e, para isto, haveria mais custo. Porém, se tem como objetivo o avanço dentro do aplicativo, partindo nesta perspectiva.

Em relação aos comentários reportados pelos especialistas referente ao aplicativo MeduLar sem Medo^®^, estes ratificam a validação com excelência, tais como, excelente ferramenta de suporte rápido e fácil à pessoa com LM, contribuindo para o esclarecimento de dúvidas e, ainda, proporcionando a identificação no que poderá melhorar no cuidado; recursos necessários para atuação ativamente do autocuidado, auxiliando na adesão do tratamento, com fatores que promovam mudanças de hábitos e, consequentemente, a prevenção de complicações. A sua divulgação na Atenção Primária e serviços de saúde poderá acrescentar possibilidade ao enfermeiro na melhoria e acompanhamento do processo de aprendizagem da pessoa com LM.

## Discussão

O aplicativo MeduLar sem Medo^®^ foi validado por especialistas com IVC global 0,98, o que corresponde à excelente validade de conteúdo^15^, indicando que os especialistas estão em concordância estatisticamente significante sobre o conteúdo do aplicativo móvel. 

Tal fato aponta que o aplicativo revela a compreensão do tema proposto, segue uma sequência lógica e incorpora de forma ordenada a organização do material e a técnica do cateterismo intermitente limpo, o que viabiliza esclarecer dúvidas. Ademais, proporciona informações de conteúdo acurado, claro e esclarecedor, além de ser interativo, podendo despertar o interesse do usuário pelo tema. 

Logo, a validação pelos especialistas com vasta experiência, além de formação acadêmica significante, possibilitou a adequação, o aprimoramento do aplicativo e avaliação criteriosa e de credibilidade da tecnologia para a versão final. Além disso, a atuação dos especialistas de diferentes regiões permite a adaptação do produto construído para o contexto geral brasileiro, considerando a diversidade cultural das dimensões continentais.

Quanto à ocupação profissional, houve predominância de enfermeiros atuantes diretamente com a assistência na temática, ou seja, que assistem diretamente a pessoa com LM, conhecendo as fragilidades apresentadas no cuidado. Destaca-se que, baseando-se neste estudo, foi baixa a participação de especialistas com publicação de artigos sobre a temática e que pertenciam a grupos de pesquisa na temática. 

Ademais, o Módulo I - Objetivos mostrou potencial para o processo ensino-aprendizagem, pois poderá possibilitar reflexão à pessoa com LM e incentivar mudança de hábitos. Estudiosos avaliaram um outro aplicativo móvel, tomando por base a experiência de gestante, acompanhadas em consultas pré-natal no setor público de saúde. Observou-se que o aplicativo foi uma ferramenta favorável para auxiliar no aprimoramento e na construção de novos conhecimentos e melhorar o autocuidado, quando incentivado seu uso por profissional^17^.

Em outro estudo, os autores desenvolveram e validaram o aplicativo móvel voltado para gestantes adolescentes da atenção primária à saúde. O aplicativo foi avaliado positivamente, tanto na qualidade quanto na usabilidade, através do qual concluíram que a criação do recurso digital se apresenta como forma complementar e de suporte ao processo de educação às gestantes^18^. Similarmente, outro aplicativo móvel sobre os cuidados de enfermagem às pessoas com diabetes mellitus mostrou potencial para auxiliar o profissional enfermeiro no rastreamento, no acompanhamento e na prevenção de complicações nos usuários com diabetes^19^.

Outrossim, ao analisar a eficácia de intervenção liderada por enfermeiras, constatou-se os benefícios do aplicativo móvel para mães com depressão. A intervenção foi eficaz e o aplicativo de fácil usabilidade foi considerado complemento aos serviços existentes de apoio para mães com depressão^20^.

Por conseguinte, as evidências de pesquisas com propostas semelhantes corroboram o quanto um aplicativo tem potencial como ferramenta educativa e incentivadora ao autocuidado. Nesta perspectiva, o Módulo II, Conteúdo, mostrou-se esclarecedor e correto quanto às informações. Assim, o produto apresenta informações científicas baseada em revisão integrativa e com referencial teórico sob atenção singular para a pessoa com LM. Destaca-se que a adesão ao autocuidado é complexa, a reabilitação requer domínio de conhecimentos técnicos específicos e entendimento do enfermeiro das fases de aceitação pelo paciente da própria condição.

Isto é, o domínio de entendimento do profissional nas fases de negação, repercussão, ajustamento e reconstrução à nova performance, com foco na superação, interação e inclusão social é indispensável no êxito da reabilitação^21^. Ou seja, o aplicativo apresenta estratégias motivacionais aos que se encontram na fase de negação e estratégias educacionais aos que estão na fase de ajustamento e/ou reconstrução, com potencial impacto de melhoria pessoal com DNTUI. Desta forma, espera-se contribuir para melhor enfrentamento em cada fase.

Em estudo qualitativo realizado em 2020, no município de Natal, Rio Grande do Norte, Brasil, com objetivo de compreender os conhecimentos e as práticas dos profissionais da atenção primária sobre o cuidado à pessoa com LM, os autores referiram que o cuidado é permeado por dificuldades estruturais, sobretudo, com carência de capacitação dos profissionais para o cuidado integral, com destaque para necessidade de ações de educação permanente para melhoria da assistência^22^.

Assim, observa-se que o aplicativo MeduLar sem Medo^®^ tem potencial de transformação no processo ensino-aprendizagem, sendo ferramenta de complementação, pois amplia a integração do profissional com a pessoa com LM, com vista a alcançar a integralidade no cuidado, devido à carência de capacitação dos profissionais. Também constitui instrumento de educação permanente nas instituições de saúde. 

À vista disto, o enfermeiro que acompanha os progressos dos enfrentamentos e o despertar da motivação da pessoa com deficiência em cuidar de si e de reconhecer a necessidade de ajuda para o autocuidado, deve estar pronto para adentrar nesta etapa do tratamento, pois encontra espaço propício para investir na educação em saúde e colaborar na reabilitação e autonomia do paciente^21^.

Ademais, o conteúdo referente à orientação quanto ao cateter convencional, sugerido pelo especialista, foi atendido. Apesar do padrão ouro ser o cateter hidrofílico, essa não é a realidade de muitos usuários. Vale destacar que é direito garantido por lei que o indivíduo que realiza o cateterismo intermitente receba o material para realização. A Portaria nº 37, de 24 de julho de 2019, artigo 1º, estabelece o cateter hidrofílico para cateterismo intermitente em indivíduos com LM, conforme estabelecido pelo Ministério da Saúde, no âmbito do Sistema Único de Saúde (SUS). Assim, a garantia de acesso ao material necessário ao procedimento faz parte do tratamento de saúde desses indivíduos, portanto, sendo dever do SUS garantir a disponibilização. Porém, cada município possui autonomia para estabelecer o fluxo de liberação desse material^23^. Quando há dificuldades que impossibilitam o acesso do paciente a este material, é disponibilizado o cateter convencional. 

Concernente à referência ao Módulo III - Linguagem - este mostrou conter linguagem clara, acessível e interativa. Em revisão integrativa realizada, considerando o período de 2010 a 2020, para avaliar aplicativos móveis desenvolvidos e implementados em crianças e adolescentes com doenças crônicas, identificou-se que o uso do humor, da gamificação e da linguagem simples e visual atraente despertou interesse e facilitou a utilização do aplicativo e, ainda, favoreceu a adesão ao autocuidado^24^.

Sendo assim, a interação com o usuário é relevante, pois será por meio dela que o aplicativo executará o objetivo educacional e motivacional. As estratégias de interação foi a linguagem simples apresentada no formato de diálogo. Também foram utilizados recursos tecnológicos como imagens, áudios e vídeos, a fim de tornar o aplicativo mais atrativo e menos cansativo. Outra estratégia utilizada foi a gamificação que poderá auxiliar o usuário a mensurar o conhecimento adquirido. Além disso, espera-se, com a utilização de áudios informativos gravados pela pesquisadora, sensibilizar para o autocuidado.

O Módulo IV - Relevância - mostrou-se pertinente no processo de autocuidado com estratégias de reflexão e ação. Além disso, buscaram-se as principais leis que tratam dos direitos à pessoa com LM, com intuito de fornecer informações, por meio de um link que a direcionará para o site oficial.

Em relação ao Módulo V - Funcionalidade - este mostrou desempenhar função de aprendizagem à pessoa com DNTUI e, também, auxiliar o profissional que fará o processo de alta hospitalar ou o profissional que fará o acompanhamento na reabilitação^21^.

Por fim, o Módulo VI - Usabilidade - mostrou-se simples e intuitivo ao uso, além de proporcionar conhecimento de conceitos complexos os transformando em compreensivos, facilitando o entendimento da nova fisiologia urinária da pessoa com LM e as respectivas complicações^21^. 

Portanto, a proposta desta tecnologia é a inserção da pessoa com LM no próprio tratamento. A saúde é definida como “estado completo de bem-estar físico, mental e social, e não consiste apenas na ausência de doença ou enfermidade”^25^. Conforme o sistema de saúde, o profissional deve estar preparado para ouvir o usuário, compreender o contexto social em que está inserido e, a partir de então, atender as demandas e as necessidades, atentando, sobretudo, para prevenção de agravos à saúde^25^. Uma das medidas essenciais para melhorar a sobrevivência, saúde e participação da pessoa com LM é o acesso aos cuidados de saúde contínuos e à educação em saúde^26^.

Além do mais, a Portaria Nº 793 de 2012, instituiu a Rede de Cuidados à Pessoa com Deficiência, no âmbito do Sistema Único de Saúde. No capítulo I, artigo 2º, nas disposições gerais, recomenda, dentre outras diretrizes para o funcionamento, a promoção de estratégias de educação permanente, inovação tecnológica em reabilitação, articuladas às ações do Centro Nacional em Tecnologia Assistiva, uso de tecnologias livres para gestão, voltadas principalmente às áreas de informação^27^. 

Nos artigos 3º e 4º, tendo como objetivos da Rede de Cuidados, destaca-se ampliar o acesso e qualificar o atendimento às pessoas com deficiência no SUS, promover mecanismos de formação permanente para profissionais de saúde, produzir e ofertar informações sobre direitos das pessoas, medidas de prevenção e cuidado e serviços disponíveis da rede, por meio de cadernos, cartilhas e manuais^27^.

Já no capítulo II, artigo 11, apresentam-se os componentes da Rede de Cuidados que se organizam em: I- Atenção Básica; II- Atenção Especializada em Reabilitação (CER); III- Atenção Hospitalar. Cujos componentes da Rede serão articulados entre si, de forma a garantir a integralidade do cuidado e o acesso regulado a cada ponto de atenção e/ou aos serviços, quais sejam, dentre outros: manejo clínico e medidas de prevenção da perda funcional, de redução do ritmo da perda funcional e/ou da melhora ou recuperação da função^27^.

Ainda, há evidências sobre a necessidade global e o acesso a produtos assistivos. Ademais, proporciona uma série de recomendações para expandir a disponibilidade e o acesso. O relatório recomenda, dentre outros, melhoria no acesso, nos sistemas de educação, saúde e assistência social, garantia à disponibilidade, segurança, eficácia e acessibilidade dos produtos assistivos e envolvimento ativo dos usuários de tecnologia assistiva e das famílias destes^28^.

Nessa conjuntura, a tecnologia assistiva é um termo abrangente para produtos assistivos e para os sistemas e serviços relacionados. Logo, os produtos assistivos favorecem melhorar o desempenho nos principais domínios funcionais, como o autocuidado, e podem constituir-se em produtos físicos, como cadeira de rodas ou softwares e aplicativos digitais^28^.

Posto isso, o aplicativo MeduLar sem Medo^®^ apresenta qualificação especializada e tópicos de educação e motivação para contribuir com as Redes de Cuidados à Pessoa com Deficiência, no âmbito do SUS, a fim de promover a saúde, além da contribuição na capacitação dos profissionais de saúde nesta temática.

Com este estudo, espera-se contribuir com inovação de aprendizagem na assistência, na educação e no ensino. Por fim, o aplicativo móvel foi registrado no Instituto Nacional de Propriedade Industrial (INPI), sob domínio da Universidade Federal do Estado do Rio de Janeiro, com acesso gratuito ao usuário.

Como limitações do estudo, refere-se a não validação desta tecnologia pela pessoa com LM, quanto à usabilidade e à aplicabilidade, ou seja, sua repercussão na melhoria da adesão ao autocuidado, o que se configura como proposta de continuidade. E, ainda, o fato de o aplicativo não possuir armazenamento de informações em nuvem dos usuários, o que se avalia para possibilidade futura.

## Conclusão

Em síntese, o aplicativo MeduLar sem Medo^®^ foi validado por especialistas quanto ao conteúdo para orientação às pessoas com disfunção neurológica do trato urinário inferior, com excelente índice global.

Tem-se como implicações futuras a promoção da educação em saúde da pessoa com DNTUI, de modo a favorecer o autocuidado com as informações técnicas e promovendo adesão ao cuidado, por meio de estratégias efetivas e motivando mudanças de hábitos. Também considera-se colaborar com a capacitação dos profissionais e contribuir com a aprendizagem dos acadêmicos de saúde, aproximando-os a este tema precocemente. 

Afinal, como proposta futura de pesquisa, indica-se a aplicação do aplicativo móvel em populações a que se destina, vislumbrando à incorporação desta tecnologia do cuidado no âmbito do Sistema Único de Saúde por meio da Rede de Cuidados, seja na atenção primária ou especializada, de modo a auxiliar na redução de complicações secundárias e na redução de custos para o sistema de saúde, por meio da educação.

# Translation: Validación de una aplicación móvil para adultos con disfunción neurológica del tracto urinario inferior*

**DOI:** 10.1590/1518-8345.7145.4322

**Authors:** Danielle Soraya Lourenço Fernandes Gomes, Priscilla Alfradique de Souza, Gisela Maria Assis, Danielle Galdino de Paula

**Affiliations:** 1 Universidade Federal do Estado do Rio de Janeiro, Rio de Janeiro, RJ, Brasil.; 2 Instituto Nacional de Traumatologia e Ortopedia, Unidade de Estomaterapia, Rio de Janeiro, RJ, Brasil.; 3 Universidade Federal do Estado do Rio de Janeiro, Escola de Enfermagem Alfredo Pinto, Rio de Janeiro, RJ, Brasil.; 4 Universidade Federal do Paraná, Hospital de Clínicas, Curitiba, PR, Brasil.

**Keywords:** Vejiga Urinaria Neurogénica, Traumatismos de la Médula Espinal, Cateterismo Uretral Intermitente, Educación para la Salud, Aplicaciones Móviles, Estomaterapia

## Abstract

**Objetivo:**

validar con especialistas el contenido de una aplicación móvil para orientación de pacientes con disfunción neurológica del tracto urinario inferior.

**Método:**

estudio metodológico. Para la validación del contenido, participaron 81 enfermeros especialistas. El instrumento de recolección de datos se elaboró en formato de formulario electrónico y contuvo evaluaciones sobre objetivos, contenido, lenguaje, relevancia, funcionalidad y usabilidad de la aplicación. Los datos se sometieron a análisis estadístico descriptivo e inferencial, basado en la medición del Índice de Validez de Contenido.

**Resultados:**

el índice de validez de contenido global del prototipo de la aplicación fue de 0,98. El ítem más bajo (0,93) se refirió a la adecuación del tamaño de la fuente, y el módulo más alto (1) se asoció a la usabilidad, que mostró facilidad en el manejo de la aplicación. Los especialistas recomendaron corrección ortográfica y gramatical, además de adiciones y minimización de información, estandarización del lenguaje, entre otros.

**Conclusión:**

el contenido de la aplicación móvil MeduLar sin Miedo^®^ fue validado con excelencia por especialistas, pues presentó recursos importantes para la enseñanza del control de la disfunción urinaria y la adhesión a la rehabilitación. Con tales resultados, es posible vislumbrar las próximas etapas de validación, con aplicación en las poblaciones a las que se destina e incorporación de esta tecnología de cuidado en el ámbito del Sistema Único de Salud.

## Destacados

**(1)** Estudio innovador en el proceso de enseñanza-aprendizaje de enfermería.

**(2)** Innovación en el ámbito del Sistema Único de Salud.

**(3)** Herramienta educativa de fácil acceso para el usuario.

**(4)** Proporciona al enfermero el seguimiento del proceso de aprendizaje.

**(5)** Desarrollo de competencias profesionales para los estudiantes de salud.

## Introducción

La Lesión Medular (LM) es un evento impactante, ya que afecta a la persona involucrada y a la familia/cuidador. Después de la aparición de la LM, el individuo sufre alteraciones físicas y emocionales que, en grados variados, pueden ser irreversibles o fatales. Un ejemplo de condición irreversible es la disfunción neurológica del tracto urinario inferior^1^ ^)-(^ ^2^.

Según la investigación realizada en 2019, por la Red Sarah de Hospitales de Rehabilitación, mediante entrevistas a pacientes hospitalizados, los accidentes de tránsito fueron la primera causa externa de hospitalización, con el 47,7% de los casos. Las agresiones (incluyendo arma de fuego, arma blanca y agresión física) constituyen la segunda causa externa de hospitalización, con el 22,6% de los casos. También aparecen, como causas externas, en la misma investigación, las caídas, con el 15,5%; los accidentes de buceo, con el 4,5%; los impactos por objetos pesados, con el 2,7%; entre otras, con el 7,0%^3^. Y de acuerdo con la *European Association of Urology*, la prevalencia de LM traumática en países desarrollados varía de 280 a 906/millón^4^.

Sin embargo, en un estudio cuantitativo, retrospectivo y descriptivo con el objetivo de levantar el perfil epidemiológico del trauma raquimedular en el estado del Distrito Federal, Brasil, entre los años 2018 y 2020, al referirse a las complicaciones relacionadas al trauma raquimedular, las más prevalentes fueron: lesiones por presión, neumonía, atelectasia, infección del tracto urinario y espasticidad^5^.

Aún, se entiende que en personas con Disfunción Neurológica del Tracto Urinario Inferior (DNTUI), sin el manejo adecuado de la vejiga, se esperan complicaciones vesicourinarias como infección urinaria recurrente, cálculo vesical, fístula peno-uretral, reflujo vesicoureteral, hidronefrosis y pérdida de la función renal^1^ ^)-(^ ^2^.

Por lo tanto, los cuidados a las personas con disfunción urinaria son esenciales y deben ser orientados y acompañados por un profesional de salud, con el fin de crear capacidad y fortalecer el proceso de rehabilitación. Se destaca que, entre los cuidados, se debe observar el patrón urinario y sus características; señalar cambios en el organismo e incentivar al individuo a redescubrirse en su nueva condición de vida; comprender el entendimiento sobre la disfunción urinaria; orientar sobre la realización del Cateterismo Intermitente Limpio (CIL) y los cambios conductuales; identificar factores de riesgo para las complicaciones vesicourinarias; presentar alternativas temporales a la incontinencia; observar el uso de medicamentos; y reconocer la aceptación y afrontamiento de esta nueva condición^4^.

Dicho esto, investigadores realizaron un estudio en un Hospital Universitario en Minas Gerais, Brasil; e investigaron si los enfermeros que atienden a personas con LM están preparados para orientar sobre el CIL. Los autores concluyeron que la mayoría de los pacientes investigados no recibieron orientación antes del alta hospitalaria. Cabe destacar que el hospital es una referencia en la atención a la LM^6^. Es importante destacar que la asistencia de enfermería sistematizada debe promover la mejora del tratamiento del paciente y la prevención de complicaciones. De este modo, el enfermero desempeña un papel importante en la educación en salud, el proceso de rehabilitación y en la reinserción social de personas con LM^6^.

En contrapartida, el uso de tecnologías móviles en la educación en salud se ha mostrado cada vez más relevante y eficaz en la actualidad. Por medio de aplicaciones, mensajes de texto, videos y otras herramientas digitales, es posible difundir información y orientaciones sobre cuidados de salud de manera accesible e interactiva. Este enfoque permite alcanzar a un mayor número de personas, independientemente de su ubicación geográfica, proporcionando un mayor compromiso en la gestión de la salud. Además, las tecnologías móviles permiten la personalización del contenido según las necesidades de cada usuario, haciendo el proceso de aprendizaje más eficaz y adaptado a las particularidades. Por lo tanto, la integración entre educación en salud y tecnologías móviles representa una herramienta poderosa para el enfermero en el proceso de rehabilitación.

Actualmente, no hay aplicaciones móviles (APP) referidas en la literatura nacional e internacional que orienten sobre el manejo adecuado de la DNTUI, tan solo se encuentra cómo fuente de información para pacientes con LM: folletos, infografías, videos y manuales. Todavía son pocas las herramientas educativas digitales desarrolladas y validadas para colaborar en el proceso de enseñanza-aprendizaje^7^. Se presenta, por lo tanto, como un potencial innovador, ya que se trata de una herramienta facilitadora en el proceso de enseñanza-aprendizaje para los usuarios, profesionales y académicos.

En vista de esto, una investigación global sobre salud digital propone nuevos horizontes para la salud y ratifica que las tecnologías móviles están cada vez más presentes en la vida de las personas y avanzan de forma dinámica y veloz en la sociedad, constituyendo nuevas relaciones educacionales^8^. O sea, el uso y la ampliación de soluciones digitales de salud pueden innovar la forma en que las personas en el mundo alcanzan estándares más altos de salud y acceden a servicios y/o informaciones para promover y proteger la salud^9^. Además, se destaca que el fortalecimiento en salud digital forma parte de las prioridades de la Estrategia de Salud Digital para Brasil (2020-2028) (ESD28)^9^. Algunos objetivos de la ESD28 consisten en: apoyo a la mejora de la atención en salud, compromiso del usuario como protagonista de su propia salud, entorno de conectividad en salud y ecosistema de innovación^9^.

Al considerar que las tecnologías móviles pueden ser eficientes para la promoción de la salud en personas con DNTUI y que no hay aplicaciones disponibles en la literatura sobre orientación a personas con disfunción neurológica del tracto urinario inferior, se propuso el presente estudio. Así, se objetivó validar el contenido de una aplicación móvil por especialistas en enfermería, con miras a la orientación de pacientes adultos con disfunción neurológica del tracto urinario inferior.

## Método

### Tipo de estudio

Este estudio forma parte del proyecto titulado “MeduLar sin Miedo^®^: aplicación móvil para adultos con disfunción neurológica del tracto urinario inferior secundaria a lesión medular traumática”. Y se trata de un estudio metodológico, en la modalidad de innovación tecnológica para la validación de una aplicación educativa.

Además, el marco de referencia utilizado fue el *Design Science Research* (DSR), que se desarrolla en seis etapas: se identificó el problema/motivación; a partir de esto, se enumeraron los objetivos; se desarrolló el producto; se ejecutó el proceso de prueba; se realizó la evaluación experimental mediante la validación por especialistas en el área; y se finalizó con la propuesta de divulgación científica, con el fin de que sea aplicado en situaciones similares por diversas organizaciones^10^.

El DSR es una metodología centrada en la resolución de problemas identificados en el contexto práctico, a partir de nuevos conocimientos científicos aplicados a la producción de productos, generalmente de naturaleza tecnológica^10^. Esta metodología tiene como núcleo directrices tales como: el objeto de investigación debe ser un producto; el problema abordado debe ser relevante; y el producto debe ser útil para el usuario. Y, aún, requiere una evaluación rigurosa del producto, siguiendo métodos sistemáticos de investigación; contribuciones innovadoras al área de conocimiento de la investigación; uso adecuado de los recursos para alcanzar las metas deseadas; y comunicación de los resultados de la investigación a los usuarios^10^.

En este ínterin, las tres etapas iniciales orientadas por el DSR se desarrollaron en un estudio anterior, también permeado por una revisión integrativa de la literatura^11^, con análisis de evidencias científicas como contenido de la aplicación, guiado por el marco de referencia de la Teoría del Déficit de Autocuidado de Orem en todo el proceso de construcción. En este artículo, se presentan la etapa de validación de contenido y la proposición del prototipo a partir de las consideraciones.

### Lugar del estudio

La recolección de datos se realizó en un entorno virtual, con cobertura nacional en las cinco regiones de Brasil e internacional (brasileños residentes en ciudades de Colombia, España y Portugal).

### Período

La recolección de datos se realizó entre el 6 de marzo y el 10 de abril de 2023.

### Población, criterios de inclusión y muestra

La población estuvo constituida por enfermeros especialistas en estomaterapia o rehabilitación. Los criterios de inclusión fueron: tener, como mínimo, título de especialista en Estomaterapia o Rehabilitación, o experiencia mínima de cinco años en la misma área. Los criterios de exclusión fueron: enfermeros que no alcanzaran, como mínimo, cinco puntos en los criterios^12^, de acuerdo con la Figura 1.

**Figura 1:** Criterios adoptados para la selección de los especialistas de este estudio. Rio de Janeiro, RJ, Brasil, 2023^12^

CRITERIOS	PONTUACIÓN
Título de Máster en Enfermería o mínimo de 5 años de actuación profesional	4
Participación en Grupo de Investigación en la temática (>1 año)	1
Publicación de artículo sobre la temática en revistas de referencia	2
Título de Doctor en Enfermería	2
Experiencia clínica o de enseñanza en la temática (>1 año)	1
Especialización concluida en Estomaterapia/Rehabilitación	3

El contacto con los especialistas se realizó primero en grupos de trabajo en el área de rehabilitación de disfunción urinaria en Brasil, mediante direcciones electrónicas (correo electrónico) y/o aplicaciones de mensajería instantánea. También se realizó, para la selección de los especialistas, un análisis del *Currículo Lattes*, disponible en la *Plataforma Lattes* del Portal del *Conselho Nacional de Desenvolvimento Científico e Tecnológico* (CNPq), utilizando para la búsqueda los términos “*incontinência urinária*”; “*reabilitação*”; “estomaterapia”, “*bexiga neurogênica*”; y “*disfunção neurológica do trato urinário inferior*”.

Además, como estrategia de reclutamiento, se aplicó el muestreo por conveniencia y bola de nieve^13^. Para la composición del panel de especialistas, se reclutaron más de 300 especialistas, de los cuales 178 aceptaron participar y 89 respondieron al cuestionario. Uno fue excluido por no haber completado todas las respuestas del formulario y, por criterio de exclusión, siete especialistas fueron excluidos por no alcanzar el mínimo de 5 puntos en los criterios^12^. Así, la muestra final estuvo constituida por 81 especialistas.

### Variables del estudio

Se eligieron las variables sociodemográficas (sexo, edad, tiempo de actuación profesional, mayor titulación académica, experiencia asistencial en la temática, lugar de actuación, publicación y participación en grupos de investigación en la temática). Las variables relacionadas con los ítems de evaluación de contenido se separaron por módulos: objetivos (Módulo I), contenido (Módulo II), lenguaje (Módulo III), relevancia (Módulo IV), funcionalidad (Módulo V), y usabilidad (Módulo VI).

### Instrumento utilizado para la recolección de datos

El instrumento de recolección de datos se elaboró en formato de formulario *online*, utilizando la herramienta *Google Forms* ^*®*^ , compuesto por siete partes: 1ª. Compuesta por una breve presentación del proyecto; 2ª. Consentimiento Informado; 3ª. Orientaciones generales sobre el llenado del formulario; 4ª. Caracterización sociodemográfica de los especialistas; 5ª. Caracterización profesional de los especialistas; 6ª. Enlace de acceso al prototipo de la aplicación con los ítems a ser evaluados; 7ª. Agradecimiento por la participación.

### Recolección de datos

Se envió el formulario a los especialistas por correo electrónico o aplicación de mensajería. En contrapartida, se proporcionaron las informaciones para el acceso al prototipo mediante un video que presentaba las pantallas, de manera que el evaluador pudiera tener una experiencia similar a la del usuario.

### Análisis de datos

Los datos fueron sometidos a análisis estadístico descriptivo e inferencial, basado en la medición del Índice de Validez de Contenido (IVC). Este índice consiste en la medición de la proporción o porcentaje de especialistas que están de acuerdo sobre los ítems del instrumento, permitiendo analizar cada ítem, así como su integralidad^14^ ^)-(^ ^15^.

Por consiguiente, para el análisis de los datos, se consideró la transformación de la escala tipo Likert^12^, en la que se atribuyeron las siguientes clasificaciones para las opciones: totalmente en desacuerdo (valor: 0), parcialmente en desacuerdo (valor: 0,25), ni de acuerdo ni en desacuerdo (valor: 0,50), parcialmente de acuerdo (valor: 0,75), y, por fín, totalmente de acuerdo (valor: 1). Para la evaluación tanto de cada ítem como del instrumento completo, los valores para el IVC se consideraron aceptables a partir de 0,50. Para cada ítem considerado aceptable, se dividió por el número de especialistas, así, obteniendo así la proporción de concordancia^15^. Y, para el análisis de la evaluación global de los especialistas, se utilizó el sumatorio de todos los IVC calculados por separado, dividido por el número total de ítems del instrumento. En los módulos, se calculó el promedio del IVC^15^.

En esta coyuntura, la concordancia de al menos el 80% indica la adecuación del contenido, siendo pertinente su permanencia en el producto. Así, para este estudio, se consideraron válidos los ítems que obtuvieron un IVC ≥ 0,80. Luego, los que obtuvieron valores inferiores a este umbral necesitarían ser excluidos o modificados para ser reevaluados^16^. Las respuestas fueron exportadas en forma de documento en el *software* de hojas de cálculo *Excel* ^*®*^ , de *Microsoft Corporation*, y los datos fueron seleccionados, agrupados y contabilizados para su análisis.

### Aspectos éticos

El proyecto fue sometido y aprobado por el Comité de Ética en Investigación, conforme a la opinión 5.476.943. A todos los participantes de la investigación se les garantizó la confidencialidad de los datos y el anonimato.

## Resultados

Del total de 81 (100%) especialistas, el 92% eran mujeres, y la edad varió de 30 a 73 años, siendo la mayor frecuencia entre 30 y 40 años. En cuanto al tiempo de actuación profesional, el 49% presentó un tiempo superior a 15 años. Respecto a la titulación académica, el 55% había cursado la especialización, y el 84% eran estomaterapeutas. En cuanto a la experiencia asistencial a pacientes con disfunción neurológica del tracto urinario inferior, el 63% tenía experiencia superior a un año. Sin embargo, solo el 17% tenía publicaciones en la temática del estudio y solamente el 23% había participado en grupos de investigación.

En cuanto a la caracterización según el lugar de actuación, se contemplaron todas las regiones de Brasil, siendo de la Región Centro-Oeste (5%), Región Norte (6%), Región Sur (10%), Región Noreste (17%) y Región Sureste (56%). Los estados con mayor número de profesionales en esta temática fueron: Rio de Janeiro (n=20/25%), São Paulo (n=16/20%), Minas Gerais (n=9/11%) y Ceará (n=7/9%); y, además de ciudades internacionales como Valencia, España; San Cristóbal de La Laguna, España; Bogotá, Colombia; y Coimbra, Portugal, según se muestra en la Tabla 1. Las ciudades internacionales se refieren a especialistas brasileños que residen en el exterior.

**Tabla 1:** Caracterización de los especialistas de la aplicación. Rio de Janeiro, RJ, Brasil, 2023

Características	n	%
**Sexo**		
Femenino	75	92
Masculino	6	8
Edad (años)		
30 a 40	34	42
41 a 50	31	38
51 a 60	12	15
>60	2	2
**Tiempo de actuación profesional (años)**		
<5	1	1
5-10	21	26
11-15	19	23
>15	40	49
**Mayor titulación académica**		
Especialización	45	55
Maestría	19	23
Doctorado	15	18
Postdoctorado	2	2
**Experiencia asistencial en la temática**		
No	25	31
Sí < 1 año	5	6
Sí > 1 año	51	63
**Lugar de actuación por macrorregión**		
Norte	5	6
Noreste	14	17
Sureste	45	56
Centro-Oeste	4	5
Sur	8	10
Colombia	1	1
Portugal	1	1
España	2	2
**Publicación en la temática del estudio**		
No	67	83
Sí	14	17
**Participación en grupo de investigación temático**		
No	62	76
Sí > 1 año	19	23
Total	81	100

Acerca de la evaluación de la aplicación móvil por los especialistas, el índice de validez de contenido por ítems varió entre 0,93 y 1. El ítem más bajo (0,93) se refirió a la adecuación del tamaño de la fuente en la aplicación, ya que los especialistas consideraron la fuente pequeña y sugirieron aumentarla. Ya la puntuación máxima (1) se presentó en el 44% de los ítems evaluados, teniendo el Módulo II, Contenido, la mayor concentración de esta puntuación, como se muestra en la Tabla 2.

**Tabla 2:** Índice de validez de contenido de los dominios evaluados de la aplicación. Rio de Janeiro, RJ, Brasil, 2023

Módulos	IVC*
Módulo I - Objetivos	
Ítem 1- El prototipo de la aplicación contempla el objetivo propuesto	0,97
Ítem 2- El prototipo de la aplicación es adecuado para el proceso de enseñanza-aprendizaje	0,98
Ítem 3- El prototipo de la aplicación aclara dudas sobre el tema abordado	1
Ítem 4- El contenido del prototipo de la aplicación permite la comprensión del tema abordado	1
Ítem 5- El prototipo de la aplicación proporciona reflexión al público objetivo sobre el tema	0,98
Ítem 6- El prototipo de la aplicación incentiva el cambio de hábitos	0,97
Ítem 7- El contenido presentado en el prototipo de la aplicación corresponde a los objetivos propuestos en el trabajo	0,97
IVC* Módulo I	0,98
Módulo II- Contenido	
Ítem 8- El contenido sigue una secuencia lógica	1
Ítem 9- El contenido incorpora, de forma ordenada, todos los pasos para la técnica del CIL^†^	1
Ítem 10- El contenido dispone de todos los materiales necesarios para la orientación del CIL^†^	1
Ítem 11- La información que presenta el prototipo de la aplicación es correcta	1
Ítem 12- La información que presenta el prototipo de la aplicación es clara	1
Ítem 13- La información que presenta el prototipo de la aplicación es esclarecedora	1
Ítem 14- Las imágenes y los videos del prototipo de la aplicación ilustran de forma clara los contenidos presentados	0,97
Ítem 15- El tamaño de la fuente del texto es adecuado	0,93
IVC* Módulo II	0,99
Módulo III- Lenguaje	
Ítem 16- El prototipo de la aplicación presenta un lenguaje claro y accesible	0,96
Ítem 17- El prototipo de la aplicación presenta un lenguaje objetivo	0,98
Ítem 18- El prototipo de la aplicación tiene un lenguaje interactivo, permitiendo el desarrollo activo en el proceso educativo	0,97
IVC* Módulo III	0,97
Módulo IV- Relevancia	
Ítem 19- El prototipo de la aplicación contribuye al conocimiento de la disfunción urinaria	0,98
Ítem 20- El prototipo de la aplicación despierta interés en el tema	1
Ítem 21- El prototipo de la aplicación es relevante para que el usuario pueda realizar el autocuidado de forma segura	0,98
IVC* Módulo IV	0,99
Módulo V- Funcionalidad	
Ítem 22- El prototipo de la aplicación se presenta como una herramienta adecuada para el objetivo al que se destina	0,98
Ítem 23- El prototipo de la aplicación es adecuado para el público destinado	0,98
Ítem 24- El prototipo de la aplicación puede generar resultados positivos en el proceso de enseñanza-aprendizaje en la temática	0,98
IVC* Módulo V	0,98
Módulo VI- Usabilidad	
Ítem 25- El prototipo de la aplicación es fácil de usar	1
Ítem 26- El prototipo de la aplicación tiene una comprensión fácil de los conceptos teóricos utilizados y sus aplicaciones	1
Ítem 27- El prototipo de la aplicación permite que el usuario tenga facilidad en aplicar las orientaciones proporcionadas	1
IVC* Módulo VI	1
IVC* Global	0,98

*IVC= Índice de Validez de Contenido; ^†^CIL= Cateterismo Intermitente Limpio

El IVC global del prototipo de la aplicación fue de 0,98. Los ítems utilizados para el IVC se distribuyeron en los seis módulos de la siguiente manera:

1. Objetivos, validado en los siete ítems (IVC = 0,98), así, demostrando el alcance de la meta en relación con el proceso de enseñanza-aprendizaje, abarcando aclaración de dudas, reflexión sobre el tema e incentivo a los cambios de hábitos.
2. Contenido, validado en los ocho ítems (IVC = 0,99), significando una base científica presentada de manera clara y esclarecedora.
3. Lenguaje, validado en los tres ítems (IVC = 0,97), fue el módulo con menor puntuación, pues presentó sugerencias sobre el uso de términos técnicos. Sin embargo, mencionaron claridad, objetividad, accesibilidad e interactividad.
4. Relevancia, validado en los tres ítems (IVC = 0,99), fue considerado relevante para el usuario.
5. Funcionalidad, validado en los tres ítems (IVC = 0,98), representando adecuación al público objetivo y siendo considerada una herramienta para la educación del paciente.
6. Usabilidad, validado en los tres ítems (IVC = 1), este módulo presentó la mayor puntuación, expresando la comprensibilidad en el uso, la fácil asimilación de los conceptos teóricos, visto que son técnicos y complejos, además de proporcionar la facilidad en la aplicación de las orientaciones proporcionadas.

Es importante mencionar que los especialistas que validaron el contenido recomendaron correcciones ortográficas y gramaticales, además de adiciones y minimización de informaciones y estandarización del lenguaje. Además, los participantes - que respondieron a las preguntas objetivas del instrumento - hicieron sugerencias y comentarios sobre la aplicación. Las sugerencias fueron atendidas en función de su congruencia con la literatura, como se muestra en la Figura 2.

**Figura 2:** Síntesis de las sugerencias y comentarios de los especialistas. Rio de Janeiro, RJ, Brasil, 2023

Sugerencias	Atendida
Agregar técnica de cateterismo con catéter convencional.	Sí
Habilitar aplicación para comando de voz.	Sí
Sustituir términos técnicos.	Sí
Reducción de texto y aumento de imagen.	Sí
Gamificación para mayor interactividad.	Sí
Incluir vivencia social.	Sí
Probar con el usuario.	No
Manejo del intestino neurógeno.	No
Algo práctico para registrar escalas y diarios directamente en la aplicación.	No

Las sugerencias consideradas pertinentes condujeron a alteraciones, correcciones y mejoras de la aplicación. Otras, por no adecuarse al objetivo propuesto, no generaron modificaciones. Se enfatiza que la validación por especialistas ocurrió en un solo momento, no habiendo necesidad de reevaluación de ítems. 

Se procedió con las sugerencias específicas, de las cuales la gran mayoría fueron atendidas. El Módulo I, referente al objetivo, recibió el mayor número de sugerencias, con el propósito de ser más dinámico, interactivo y con más información, incluyendo más imágenes, videos e instrucciones con grabaciones en la voz de la autora.

Además, se sugirió habilitar el comando de voz en la aplicación para usuarios analfabetos o con agudeza visual comprometida. A partir de la sugerencia de los especialistas, se realizó una revisión del lenguaje y se produjeron nuevas imágenes y audios que sustituyeron los textos. 

Aún, para mejorar la interactividad con el usuario, se construyó una estrategia de gamificación, mediante un *quiz* categorizado por temas, proporcionando al usuario una herramienta de evaluación del conocimiento. Con el propósito de incentivar cambios de hábitos y de reintegrar a la sociedad, también se incluyeron en la aplicación MeduLar sin Miedo^®^ temas sobre estímulos para la convivencia social. 

En cuanto a la sugerencia de probar con el usuario, a pesar de ser importante, no se definió como parte de esta etapa de la investigación e integrará estudios futuros. Tampoco se atendieron, para este momento, registros directamente en la aplicación, ya que para esta función se necesitaría almacenamiento en la nube y, para esto, habría más costos. Sin embargo, se tiene como objetivo avanzar dentro de la aplicación partiendo de esta perspectiva.

Con relación a los comentarios reportados por los especialistas sobre la aplicación MeduLar sin Miedo^®^, estos ratifican la validación con excelencia. Se destacan aquí:

- Ser una excelente herramienta de soporte rápido y fácil para la persona con LM, contribuyendo al esclarecimiento de dudas y proporcionando la identificación de aspectos que pueden mejorar en el cuidado.
- Poseer recursos necesarios para actuar activamente en el autocuidado, auxiliando en la adhesión al tratamiento, con factores que promuevan cambios de hábitos y, consecuentemente, la prevención de complicaciones.
- Divulgar en la Atención Primaria y servicios de salud, añadiendo la posibilidad de que el enfermero mejore y acompañe el proceso de aprendizaje de la persona con LM.

## Discusión

La aplicación MeduLar sin Miedo^®^ fue validada por especialistas con un IVC global de 0,98, lo que corresponde a una excelente validez de contenido^15^, indicando que los especialistas están en concordancia estadísticamente significativa sobre el contenido de la aplicación móvil. 

Este hecho demuestra que la aplicación revela una comprensión del tema propuesto, sigue una secuencia lógica e incorpora de forma ordenada la organización del material y la técnica del cateterismo intermitente limpio, lo que facilita aclarar dudas. Además, proporciona información precisa, clara y esclarecedora, y es interactiva, pudiendo despertar el interés del usuario por el tema.

Luego, la validación por parte de especialistas con amplia experiencia y una formación académica significativa permitió la adecuación, el perfeccionamiento de la aplicación y una evaluación criteriosa y creíble de la tecnología en su versión final. Además, la actuación de los especialistas de diferentes regiones permite la adaptación del producto construido al contexto general brasileño, considerando la diversidad cultural de las dimensiones continentales.

En cuanto a la ocupación profesional, hubo predominancia de enfermeros directamente involucrados en la asistencia en la temática, es decir, que asisten directamente a la persona con LM, conociendo las fragilidades presentadas en el cuidado. Se destaca que, basándose en este estudio, fue baja la participación de especialistas con publicaciones de artículos sobre la temática y que pertenecían a grupos de investigación.

Además, el Módulo I - Objetivos, mostró tener potencial en el proceso de enseñanza-aprendizaje, además de proporcionar reflexión a la persona con LM e incentivar el cambio de hábitos. Estudiosos evaluaron una otra aplicación móvil, basada en la experiencia de gestantes, acompañadas en consultas prenatales en el sector público de salud. Se observó que la aplicación fue una herramienta favorable para auxiliar en el perfeccionamiento y en la construcción de nuevos conocimientos, mejorando el autocuidado, cuando su uso es incentivado por un profesional^17^.

En otro estudio, los autores desarrollaron y validaron una aplicación móvil dirigida a gestantes adolescentes de la atención primaria a la salud. La aplicación fue evaluada positivamente, tanto en calidad como en usabilidad, a través del cual concluyeron que la creación del recurso digital se presenta como una forma complementaria y de soporte al proceso de educación de gestantes^18^. De modo similar, otra aplicación móvil sobre los cuidados de enfermería a personas con diabetes mellitus demostró potencial para auxiliar al profesional enfermero en el rastreo, en el seguimiento y en la prevención de complicaciones en usuarios con diabetes^19^.

De igual modo, al analizar la eficacia de una intervención liderada por enfermeras se constataron los beneficios de la aplicación móvil para madres con depresión. La intervención fue eficaz y la aplicación de fácil usabilidad y se consideró un complemento a los servicios existentes de apoyo para madres con depresión^20^.

Por consiguiente, las evidencias de investigaciones con propuestas semejantes corroboran cuánto una aplicación tiene potencial para ser una herramienta educativa y de incentivo al autocuidado. En esta perspectiva, el Módulo II, Contenido, demostró ser esclarecedor y correcto en cuanto a la información. Así, el producto presenta información científica basada en una revisión integrativa y con un referencial teórico con atención singular a la persona con LM. Se destaca que la adhesión al autocuidado es compleja, la rehabilitación requiere dominio de conocimientos técnicos específicos y el entendimiento del enfermero de las fases de aceptación por parte del paciente de su propia condición.

Es decir, el dominio de comprensión del profesional en las fases de negación, repercusión, ajuste y reconstrucción hacia un nuevo desempeño, con un enfoque en la superación, interacción e inclusión social, es indispensable para el éxito de la rehabilitación^21^. O sea, la aplicación tiene estrategias motivacionales para aquellos en la fase de negación y estrategias educativas para aquellos en la fase de ajuste y/o reconstrucción, con un impacto potencial de mejora para la persona con DNTUI. Así, se espera contribuir a un mejor enfrentamiento en cada fase.

En un estudio cualitativo realizado en 2020 en Natal, Rio Grande do Norte, Brasil, con el objetivo de comprender los conocimientos y prácticas de los profesionales de atención primaria sobre el cuidado de personas con LM, los autores mencionaron que el cuidado está permeado por dificultades estructurales, especialmente por la falta de capacitación de los profesionales para el cuidado integral, destacando la necesidad de acciones de educación permanente para mejorar la asistencia^22^.

Así, se observa que la aplicación MeduLar sin Miedo^®^ tiene potencial de transformación en el proceso de enseñanza-aprendizaje, siendo una herramienta complementaria, pues amplía la integración del profesional con la persona con LM, con vistas a alcanzar la integralidad en el cuidado, debido a la falta de capacitación de los profesionales. También constituye un instrumento de educación permanente en las instituciones de salud.

En vista de esto, el enfermero que acompaña los progresos de los enfrentamientos y el despertar de la motivación de la persona con discapacidad para cuidar de sí misma y de reconocer la necesidad de ayuda para el autocuidado, debe estar preparado para entrar en esta etapa del tratamiento, pues encuentra espacio propicio para invertir en la educación en salud y colaborar en la rehabilitación y autonomía del paciente^21^.

Además, el contenido referente a la orientación sobre el catéter convencional, sugerido por el especialista, fue atendido. A pesar de que el estándar oro es el catéter hidrofílico, esa no es la realidad de muchos usuarios. Es importante poner de relieve que es un derecho garantizado por ley que el individuo que realiza el cateterismo intermitente reciba el material para realizarlo. La Portaria nº 37, de 24 de julio de 2019, artículo 1º, establece el catéter hidrofílico para cateterismo intermitente en individuos con LM, según lo establecido por el Ministerio de Salud, en el ámbito del Sistema Único de Salud (SUS). Así, la garantía de acceso al material necesario para el procedimiento forma parte del tratamiento de salud de estos individuos, por tanto, siendo deber del SUS garantizar su disponibilidad. Sin embargo, cada municipio tiene autonomía para establecer el flujo de liberación de este material^23^. Cuando hay dificultades que impiden el acceso del paciente a este material, entonces, se proporciona el catéter convencional.

En cuanto al Módulo III - Lenguaje - este mostró contener un lenguaje claro, accesible e interactivo. En una revisión integrativa realizada, considerando el período de 2010 a 2020, para evaluar aplicaciones móviles desarrolladas e implementadas en niños y adolescentes con enfermedades crónicas, se identificó que el uso del humor, la gamificación y el lenguaje simple y visualmente atractivo despertaron interés y facilitaron el uso de la aplicación, además de favorecer la adhesión al autocuidado^24^.

Así que, la interacción con el usuario es relevante, pues será por medio de ella que la aplicación ejecutará el objetivo educacional y motivacional. Las estrategias de interacción fueron el lenguaje simple, presentado en formato de diálogo. También se utilizaron recursos tecnológicos como imágenes, audios y videos, con el fin de hacer la aplicación más atractiva y menos cansadora. Otra estrategia utilizada fue la gamificación que podría ayudar al usuario a medir el conocimiento adquirido. Además, se espera, con el uso de audios informativos grabados por la investigadora, sensibilizar sobre el autocuidado.

El Módulo IV - Relevancia - mostró ser pertinente en el proceso de autocuidado con estrategias de reflexión y acción. Además, se buscaron las principales leyes que tratan los derechos de la persona con LM, con el fin de proporcionar información, a través de un enlace que la dirigirá al sitio oficial.

En relación con el Módulo V - Funcionalidad - este demostró desempeñar la función de aprendizaje a la persona con DNTUI y también de asistencia al profesional que realizará el proceso de alta hospitalaria o el profesional que realizará el seguimiento en la rehabilitación^21^.

Por fin, el Módulo VI - Usabilidad - se mostró simple e intuitivo en el uso, además de proporcionar conocimiento de conceptos complejos convirtiéndolos en comprensible, facilitando la comprensión de la nueva fisiología urinaria de la persona con LM y las respectivas complicaciones^21^.

Por lo tanto, la propuesta de esta tecnología es la inclusión de la persona con LM en su propio tratamiento. La salud se define como “estado completo de bienestar físico, mental y social, y no consiste solo en la ausencia de enfermedad o dolencia”^25^. Conforme al sistema de salud, el profesional debe estar preparado para escuchar al usuario, comprender el contexto social en el que está inserto y, a partir de ahí, atender a las demandas y las necesidades, prestando especial atención a la prevención de agravios a la salud^25^. Una de las medidas esenciales para mejorar la supervivencia, salud y participación de la persona con LM es el acceso a cuidados de salud continuos y a la educación en salud^26^.

Además, la Portaria Nº 793 de 2012 estableció la Red de Cuidados a la Persona con Discapacidad, en el ámbito del Sistema Único de Salud. En el capítulo I, artículo 2º, en las disposiciones generales, establece, entre otras directrices para el funcionamiento, la promoción de estrategias de educación permanente, innovación tecnológica en rehabilitación, articuladas a las acciones del Centro Nacional en Tecnología Asistiva, uso de tecnologías libres para gestión, enfocadas principalmente en áreas de información^27^.

En los artículos 3º y 4º, que destacan los objetivos de la Red de Cuidados, se resalta ampliar el acceso y cualificar la atención a las personas con discapacidad en el SUS, promover mecanismos de formación permanente para profesionales de salud, producir y ofrecer información sobre derechos de las personas, medidas de prevención y cuidado y servicios disponibles de la red, por medio de cuadernos, cartillas y manuales^27^.

En el capítulo II, artículo 11, se presentan los componentes de la Red de Cuidados que se organizan en: I - Atención Básica; II - Atención Especializada en Rehabilitación (CER); III - Atención Hospitalaria, cuyos componentes de la Red serán articulados entre sí, de forma a garantizar la integralidad del cuidado y el acceso regulado a cada punto de atención y/o a los servicios, entre otros: manejo clínico y medidas de prevención de la pérdida funcional, de reducción del ritmo de la pérdida funcional y/o de la mejora o recuperación de la función^27^.

Aún, hay evidencias sobre la necesidad global y el acceso a productos asistivos. Además, proporciona una serie de recomendaciones para expandir la disponibilidad y el acceso. El informe recomienda, entre otros, mejorar el acceso, en los sistemas de educación, salud y asistencia social, garantizar la disponibilidad, seguridad, eficacia y accesibilidad de los productos asistivos y el involucramiento activo de los usuarios de tecnología asistiva y de las familias de estos^28^.

En esta coyuntura, la tecnología asistiva es un término abarcador para productos asistivos y para los sistemas y servicios relacionados. Luego, los productos asistivos son capaces de mejorar el desempeño en los principales dominios funcionales, como el autocuidado, y pueden ser productos físicos, como sillas de ruedas o *softwares* y aplicaciones digitales^28^.

Dicho esto, la aplicación MeduLar sin Miedo^®^ presenta una cualificación especializada y tópicos de educación y motivación para contribuir con las Redes de Cuidados a la Persona con Discapacidad, en el ámbito del SUS, a fin de promover la salud, además de la contribución en la capacitación de los profesionales de salud en esta temática.

Con este estudio, se espera contribuir con innovación de aprendizaje en la asistencia, la educación y la enseñanza. Finalmente, la aplicación móvil fue registrada en el Instituto Nacional de Propiedad Industrial (INPI), bajo dominio de la *Universidade Federal do Estado de Rio de Janeiro*, con acceso gratuito para el usuario.

Como limitaciones del estudio, se refiere a la no validación de esta tecnología por la persona con LM, en cuanto a usabilidad y a la aplicabilidad, es decir, al impacto en la mejora de la adhesión al autocuidado, lo que se configura como propuesta de continuidad. Además, el hecho de que la aplicación no posea almacenamiento de información en la nube de los usuarios, lo que se evalúa para posibilidad futura.

## Conclusión

La aplicación MeduLar sin Miedo^®^ fue validada por especialistas en cuanto al contenido para orientación a personas con disfunción neurológica del tracto urinario inferior, mostrando un excelente índice global, lo que demuestra precisión para la población objetivo.

Como implicaciones futuras, se espera promover la educación en salud de las personas con DNTUI, de modo que favorezca el autocuidado con información técnica y promueva la adhesión al cuidado, mediante estrategias que motiven cambios de hábitos. También se considera colaborar con la capacitación de los profesionales y contribuir al aprendizaje de los académicos de salud, acercándolos a este tema desde una etapa temprana.

Al final, como propuesta futura de investigación, se indica la utilización del aplicativo móvil en las poblaciones a las que está destinado, contemplando la incorporación de esta tecnología del cuidado en el ámbito del Sistema Único de Salud por medio de la Red de Cuidados, ya sea en la atención primaria o especializada, con el objetivo de ayudar a reducir complicaciones secundarias y en la disminución de los costos para el sistema de salud mediante la educación.
